# House fly larval grazing alters dairy cattle manure microbial communities

**DOI:** 10.1186/s12866-021-02418-5

**Published:** 2021-12-15

**Authors:** Saraswoti Neupane, Christopher Saski, Dana Nayduch

**Affiliations:** 1 Department of Entomology, Kansas State University, Manhattan, KS USA; 2 Department of Plant and Environmental Sciences, Clemson University, Clemson, SC USA; 3 USDA-ARS, Center for Grain and Animal Health Research, Arthropod-Borne Animal Diseases Research Unit, Manhattan, KS USA

**Keywords:** House fly larvae, Grazing, Manure, Bacteria, Archaea, Protist, Microbial community, Diversity

## Abstract

**Background:**

House fly larvae (*Musca domestica* L.) require a live microbial community to successfully develop. Cattle manure is rich in organic matter and microorganisms, comprising a suitable substrate for larvae who feed on both the decomposing manure and the prokaryotic and eukaryotic microbes therein. Microbial communities change as manure ages, and when fly larvae are present changes attributable to larval grazing also occur. Here, we used high throughput sequencing of 16S and 18S rRNA genes to characterize microbial communities in dairy cattle manure and evaluated the changes in those communities over time by comparing the communities in fresh manure to aged manure with or without house fly larvae.

**Results:**

Bacteria, archaea and protist community compositions significantly differed across manure types (e.g. fresh, aged, larval-grazed). Irrespective of manure type, microbial communities were dominated by the following phyla: Euryarchaeota (Archaea); Proteobacteria, Firmicutes and Bacteroidetes (Bacteria); Ciliophora, Metamonanda, Ochrophyta, Apicomplexa, Discoba, Lobosa and Cercozoa (Protists). Larval grazing significantly reduced the abundances of Bacteroidetes, Ciliophora, Cercozoa and increased the abundances of Apicomplexa and Discoba. Manure aging alone significantly altered the abundance bacteria (*Acinetobacter*, *Clostridium*, *Petrimonas*, *Succinovibro*), protists (*Buxtonella*, *Enteromonas*) and archaea (*Methanosphaera* and *Methanomassiliicoccus*). Larval grazing also altered the abundance of several bacterial genera (*Pseudomonas*, *Bacteroides*, *Flavobacterium*, *Taibaiella*, *Sphingopyxis*, *Sphingobacterium*), protists (*Oxytricha*, *Cercomonas*, *Colpodella*, *Parabodo*) and archaea (*Methanobrevibacter* and *Methanocorpusculum*). Overall, larval grazing significantly reduced bacterial and archaeal diversities but increased protist diversity. Moreover, total carbon (TC) and nitrogen (TN) decreased in larval grazed manure, and both TC and TN were highly correlated with several of bacterial, archaeal and protist communities.

**Conclusions:**

House fly larval grazing altered the abundance and diversity of bacterial, archaeal and protist communities differently than manure aging alone. Fly larvae likely alter community composition by directly feeding on and eliminating microbes and by competing with predatory microbes for available nutrients and microbial prey. Our results lend insight into the role house fly larvae play in shaping manure microbial communities and help identify microbes that house fly larvae utilize as food sources in manure. Information extrapolated from this study can be used to develop manure management strategies to interfere with house fly development and reduce house fly populations.

**Supplementary Information:**

The online version contains supplementary material available at 10.1186/s12866-021-02418-5.

## Background

Cattle feces (manure) contains undigested fibers, carbohydrates, fatty acids, proteins, minerals and vitamins [1, 2] where microorganisms are prominent inhabitants [3, 4]. Diverse groups of microorganisms are involved in nutrient recycling and transfer of carbon nitrogen into higher trophic level through decomposition of complex organic matter such as cellulose, lipids, fatty acids and carbohydrates into low molecular weight compounds (or digestible compounds) such as proteins and sugars that can be utilized as carbon and energy source by diverse organisms [5]. Several species of muscid flies feed and breed in decaying organic matter as their adults require protein, sugar and water for their survival and reproduction [6, 7]. Also, for their successful development, several muscid fly larvae, such as stable fly (*Stomoxys calcitrans* L.) and house fly (*Musca domestica* L.), require live microorganisms in their diet [8, 9]. Cattle manure which is rich in both microorganisms and essential nutrients (or organic matter) can serve as a developmental substrate for muscid flies [1, 4, 10].

The house fly, a synanthropic muscid fly, completes its life cycle in a wide range of microbe rich habitats including garbage, animal/human feces and decaying organic matter [1, 6, 7]. Cattle manure harbors both prokaryotic and eukaryotic microorganisms [3, 4] and serves as an optimal substrate for house fly larval growth and development [1]. Although house fly larval survival and development requires live microbes [9, 11], certain microbes such as *Streptococcus sanguis*, *Lactococcus garviae*, *Escherichia coli*, and *Staphylococcus* sp. have been shown to promote the survival and growth while others such as *Providencia* sp. and *Bacillus* sp. suppress larval survival, growth and fitness [9, 11]. While the utilization of these prokaryotic microbes as a nutritional resource for house flies has been well described, information on the use of eukaryotic microbes, such as protists, as food for house fly larvae is extremely lacking.

When house fly larvae feed on microbes in the developmental substrate they effectively reduce the abundance of certain bacterial species while promoting others, resulting in changes to the microbial community composition and diversity in the substrate. In addition to live microbes, house fly larvae utilize nutrients such as nitrogen and phosphorus from manure as a resource [1], subsequently depleting those nutrients in the substrate. The consequence is alteration in nutrient levels that creates a competitive environment impacting survival and growth of various microbial communities in the manure. For example, vermicomposting using house fly larvae significantly reduced bacterial diversity, antibiotic resistance genes, and changed the abundances of different bacterial communities and their composition in swine manure [12, 13]. However, our understanding is limited regarding the extent that house fly larval grazing in dairy cattle manure influences the manure microbial communities, their compositions and diversity.

In this study, we aimed to i) characterize bacterial, archaeal and protist communities in dairy cattle manure used as house fly larval developmental substrate, ii) evaluate the effects of both manure aging and house fly larval grazing on microbial (bacterial, archaeal and protist) diversity and community structure, iii) assess the influence of house fly larval grazing on manure quality and, iv) evaluate the role of manure quality on microbial communities, community composition and diversity.

## Results

High throughput sequencing of 16S and 18S rRNA genes was utilized to characterize bacterial, archaeal and protist communities in the dairy cattle manure (fresh, aged and larval grazed (hereafter grazed)) and evaluated the effect of house fly larval grazing and age of manure in those communities.

### Archaeal community profiles

**Fig. 1:**
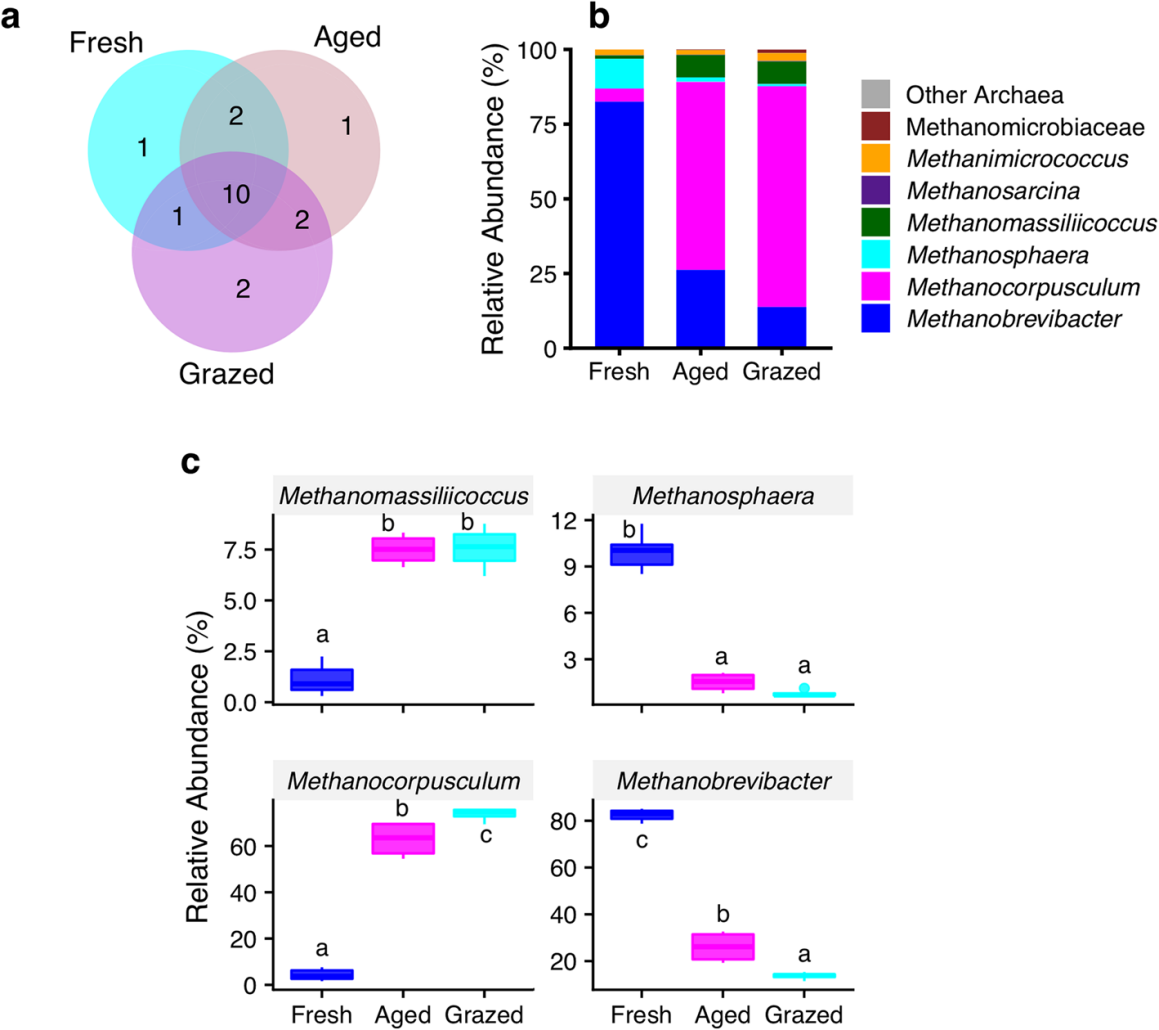
Archaeal communities in dairy cattle manure before and after aging and house fly larval grazing. **a**) Archaeal OTUs in Fresh, Aged, and house fly larval grazed (Grazed) manure types. Overlapping sections present the number of OTUs shared between and/or across manure types, and non-overlapping are unique to the manure type. Each manure type consisted of 4 replicates except fresh which included 8 replicates; **b**) Mean relative abundance of archaeal taxon at finest taxonomic resolution across manure types; **c**) Effect of manure types on the relative abundance of archaeal genera: *Methanomassiliicoccus*, *Methanosphaera*, *Methanocorpusculum*, and *Methanobrevibacter*. In the box plots, median and interquartile range (25th–75th percentiles; boxes) with upper and lower range of values are shown. The different letters on top of each box indicate the significant differences between manure types (*p* ≤ 0.05)

Archaeal communities comprised a total of 19 operational taxonomic units (OTUs). Ten out of 19 archaeal OTUs were shared among three manure types (fresh, aged, grazed manure) (Fig. 1a). Shared OTUs were represented by the most abundant OTUs in each manure type which consisted of the largest proportion of the total abundance in each manure type: fresh (99.0%), aged (98.4%) and grazed (98.5%). Interestingly, all archaeal communities were classified to a single phylum, Euryarchaeota; therefore, no variation in abundance of Euryarcheaota was expected across manure types. At the family level, manure type significantly affected the abundance of Methanobacteriaceae, Methanocorpusculaceae and Methanomassiliicoccaceae. Multiple comparison of means revealed that the relative abundance of Methanobacteriaceae was significantly greater in fresh (92.5%) compared to aged (27.7%; *p* < 0.0001) and grazed (14.6%; *p* < 0.0001) (Fig. S1a) and lower in grazed compared to aged (*p* = 0.003) manure. Similarly, the relative abundance of Methanocorpusculaceae was significantly greater in both aged (62.9%; *p* < 0.0001) and grazed (73.9%; *p* < 0.0001) compared to fresh (4.38%) manure (Fig. S1b). The relative abundance of family Methanomassiliicoccaceae was significantly greater in both aged (7.5%; *p* < 0.0001) and grazed (7.6%; *p* < 0.0001) compared to fresh (1.1%; Fig. S1c), however, no difference was observed between aged and grazed (*p* = 0.99) manure.

The archaeal genera *Methanobrevibacter, Methanocorpusculum, Methanomassiliicoccus* and *Methanosphaera* (Fig. 1b, c) were dominant in manure, and manure type significantly affected the abundance of all three genera (*p* < 0.0001). The relative abundance of *Methanobrevibacter* was significantly greater in fresh (82.52%) compared to aged (26.19%; *p* < 0.0001) and grazed (13.81%; *p* < 0.0001) and lower in grazed compared to aged (*p* = 0.001) manure (Fig. 1c). The relative abundance of *Methanomassiliicoccus* was significantly greater in both aged (7.5%; *p* < 0.0001) and grazed (7.6%; *p* < 0.0001) compared to fresh (1.5%; Fig. 1c) manure. Similarly, the relative abundance of *Methanocorpusculum* was significantly higher in grazed (73.9%) compared to both fresh (4.4%; *p* < 0.0001) and aged (62.9%; *p* < 0.0001) manure (Fig. 1c). The relative abundance of *Methanosphaera* was significantly lower in both aged (1.5%, *p* < 0.0001) and grazed (0.8%; *p* < 0.0001) compared to fresh (10.0%) manure (Fig. 1c).

### Bacterial community profiles

**Fig. 2:**
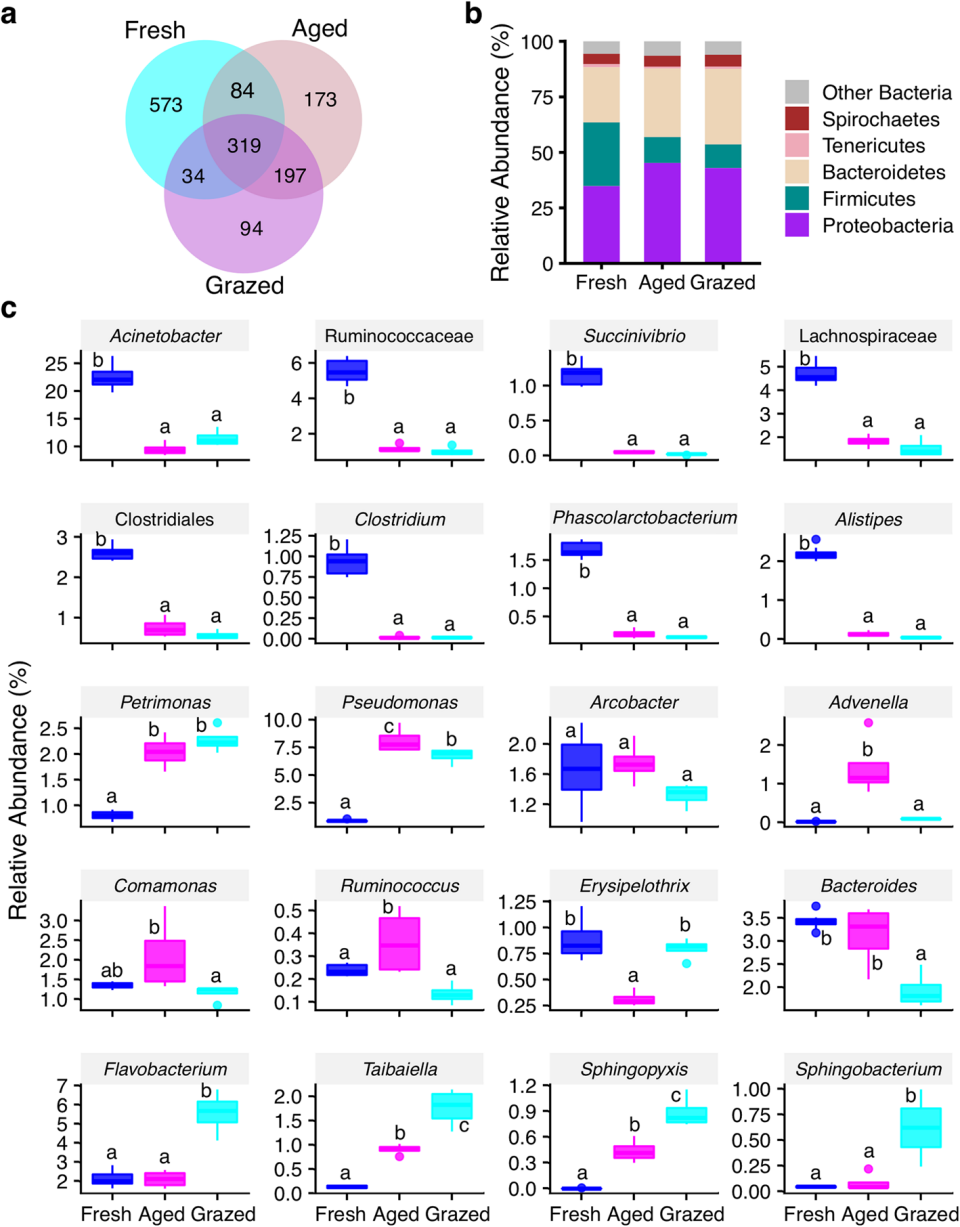
Bacterial communities in dairy cattle manure before and after aging and house fly larval grazing. **a**) Bacterial OTUs in Fresh, Aged, and house fly larval grazed (Grazed) manure types. Overlapping sections present the number of OTUs shared between and/or across manure types, and non-overlapping are unique to the manure type. Each manure type consisted of 4 replicates except Fresh which included 8 replicates; **b**) Mean relative abundance of bacterial phyla in three manure types; **c**) Effect of manure types on the relative abundance of bacterial taxa: *Acinetobacter*, Ruminococcaceae, *Succinivibrio*, Lachnospiraceae, Clostridiales, *Clostridium* sensu stricto, *Phascolarctobacterium*, *Alistipes*, *Petrimonas*, *Pseudomonas*, *Arcobacter*, *Advenella*, *Comamonas*, *Ruminococcus*, *Erysipelothrix*, *Bacteroides*, *Flavobacterium*, *Taibaiella*, *Sphingopyxis*, *Sphingobacterium*. In the box plots, median and interquartile range (25th–75th percentiles; boxes) with upper and lower range of values are shown. The different letters on top of each box indicate the significant differences between manure types (*p* ≤ 0.05)

Manure bacterial communities comprised 1474 OTUs, among them only 21.64% of OTUs were shared across the manure type (Fig. 2a). These shared OTUs represented the largest portion of the total abundance within each manure type (fresh: 75.4%, aged: 84.8% and grazed: 87.5%). At the higher taxonomic levels, manure bacterial communities were dominated by the phyla Bacteroidetes, Firmicutes and Proteobacteria. Manure type significantly influenced the abundance of all of those phyla (*p* < 0.0001). Further, the relative abundance of Bacteroidetes was significantly greater in both grazed (34.0%; *p* < 0.0001) and aged (30.9%; *p* < 0.0001) compared to fresh (24.8%; Fig. 2b), and greater in grazed compared to aged (*p* = 0.0060) manure. The relative abundance of Firmicutes was significantly lower in both grazed (10.7%, *p* < 0.0001) and aged (11.7%; *p* < 0.0001) compared to fresh manure (28.7%, Fig. 2b). Similarly, the relative abundance of Proteobacteria was significantly greater in both grazed (43.0%; *p* < 0.0001) and aged (45.2%; *p* < 0.0001) compared to fresh manure (34.8%) but there was no difference between grazed and aged manure. At the family level, manure types significantly affected the abundance of selected families (*p* < 0.0, Fig. S2), where the relative abundances of families Veillonellaceae and Succinivibrionaceae were significantly lower in grazed compared to both fresh (*p* < 0.0001 and < 0.0001 respectively) and aged (*p* = 0.0002 and 0.0003 respectively; Fig. S2 e, k). The relative abundances of the most dominant families, Moraxellaceae, Lachnospiraceae, Ruminococcaceae, Prevotellaceae, Aeromonadaceae, Clostridiaceae, Erysipelotrichaceae, were significantly lower in both aged (*p* < 0.0001, 0.0004, 0.0004, 0.0004, < 0.0001, < 0.0001, < 0.0001, respectively) and grazed (*p* < 0.0001, < 0.0001, *<* 0.0001, < 0.0001, < 0.0001, 0.0003, 0.0033, respectively) compared to fresh manure (Fig. S2 j, b, c, g, l, a, d). Interestingly, the relative abundances of families Flavobacteriaceae, Porphyromonadaceae, Sphingobacteriaceae, Comamonadaceae, Pseudomonadaceae and Xanthomonadaceae were greater in both aged (*p* = 0.0004, 0.0003, 0.0004, 0.0003, < 0.0001, *<* 0.0001, respectively) and grazed (*p* < 0.0001, < 0.0001, < 0.0001, 0.0001, 0.0004, 0.0004, respectively) compared to fresh manure (Fig. S2 h, i, m, n, o, p).

The abundance of several bacteria at the lowest taxonomic level was significantly affected by manure type (Fig. 2c, S3). Rumen-associated bacterial taxa such as *Acinetobacter*, Ruminococcaceae unclassified, *Succinivibrio*, Lachnospiraceae unclassified, Clostridiales unclassified, *Clostridium*, *Phascolarctobacterium, Alistipes* dominated the fresh manure whereas environmental bacteria such as *Petrimonas, Pseudomonas*, *Taibaiella*, Rhodobacteriaceae unclassified, *Flavobacterium*, *Sphingobacterium, Sphingopyxis* dominated the grazed manure (Fig. 2c, S3). The relative abundance of the most dominant genus *Acinetobacter* was significantly lower in aged (9.4%; *p* < 0.0001) and grazed (11.6%; *p* < 0.0001) compared to fresh (22.7%) manure (Fig. 2c, Table S2), but no difference was observed between aged and grazed manure (*p* = 0.28). The second most dominant genus *Pseudomonas* had significantly greater relative abundance in aged (8.1%; *p* < 0.0001) and grazed (6.8%; *p* < 0.0001) compared to fresh (0.9%) (Fig. 2c) manure, and a significant difference was observed between aged and grazed manure (*p* = 0.0291). The relative abundance of the genus *Flavobacterium* was significantly greater in the grazed (5.6%; *p* < 0.0001) compared to fresh (2.1%; *p* < 0.0001) and aged (2.1%; *p* < 0.0001) manure (Fig. 2c, Table S2). Similarly, the relative abundance of *Bacteroides* was significantly lower in grazed (1.9%; *p* < 0.0001) compared to both fresh (3.4%; *p* = 0.0001) and aged (3.1%; *p* = 0.0027) manure (Fig. 2c, Table S2).

### Protist community profiles

**Fig. 3:**
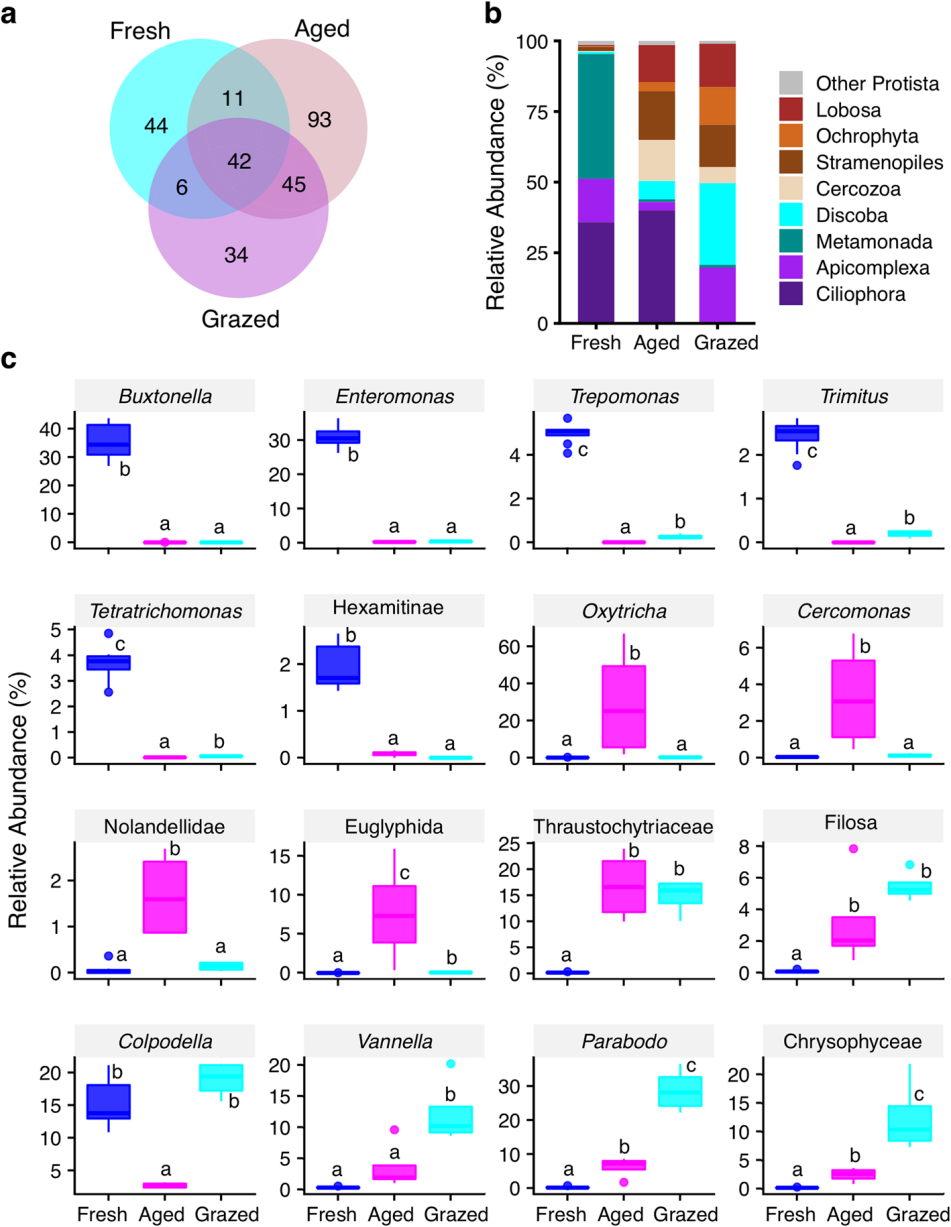
Protist communities in dairy cattle manure before and after aging and house fly larval grazing. **a**) Protist OTUs in Fresh, Aged, and house fly larval grazed (Grazed) manure types. Overlapping sections present the number of OTUs shared between and/or across manure types, and non-overlapping are unique to the manure type. Each manure type consisted of 4 replicates except fresh which included 8 replicates; **b**) Mean relative abundance of phyla in three manure types; **c**) Effect of manure types on the relative abundance of protist taxa: *Buxtonella*, *Enteromonas*, *Trepomonas*, *Trimitus*, *Tetratrichomonas*, Hexamitinae (unclassified Hexamitinae-Enteromonadida), *Oxytricha*, *Cercomonas*, unclassified Nolandellidae, unclassified Euglyphida, unclassified Thraustochytriaceae, Filosa (unclassified Filosa-Sarcomonadea), *Colpodella*, *Vannella*, *Parabodo* and unclassified Chrysophyceae. In the box plots, median and interquartile range (25th–75th percentiles; boxes) with upper and lower range of values are shown. The different letters on top of each box indicate the significant differences between manure types (*p* ≤ 0.05)

Manure protist communities consisted of 275 OTUs and only 15.27% of total OTUs were shared among manure types (Fig. 3a). These shared OTUs represented a high percentage of the total abundance within each manure type (fresh: 87.94%, aged: 88.91% and grazed: 92.83%). At the phylum level, Ciliophora, Apicomplexa, Metamonada, Discoba, Cercozoa, Lobosa, Ochrophyta and Stramenopiles were the most dominant groups (Fig. 3b). Manure type significantly affected the abundance of all groups (*p* < 0.05). Further, multiple comparison of means among manure types revealed that the relative abundance of Ciliophora was significantly lower in grazed (0.5%) compared to both fresh (35.7%; *p* = 0.002) and aged (40.0%; *p* = 0.0005) manure. However, the relative abundance of Metamonada was significantly lower in both grazed (0.9%; *p* < 0.0001) and aged (0.98%; *p* < 0.0001) compared to fresh (44.2%). The relative abundance of Discoba was significantly greater in grazed (28.9%) compared to both fresh (0.8%; *p* < 0.0001) and aged (6.6%; *p* < 0.0001) manure. Similar results were observed for Lobosa and Ochrophyta, where the relative abundances were greater in grazed (15.3 and 13.3%, respectively) and lower in both fresh (0.5 and 0.2%; *p* < 0.0001 and < 0.0001, respectively) and aged (13.1 and 3.2%; *p* = 0.26 and 0.0003, respectively) manure. The relative abundance of Apicomplexa was greater in grazed (19.2%) compared to fresh (15.4%, *p* = 0.29) and aged (2.9%; *p* < 0.0001) manure. The relative abundance of Stramenopiles was greater in both grazed (15.0%; *p* = 0.0001) and aged (17.2%; *p* < 0.0001) compared to fresh manure (1.4%).

The relative abundance of most dominant families varied among manure types (*p* < 0.05, Fig. S4). The relative abundance of Hexamitinae-Enteromonadida, Trichostomatia and Trichomonadidae were significantly reduced to very low or undetectable levels in both aged (0.9%, *p* < 0.0001; 0.0% *p* < 0.0001; and 0.0%, *p* < 0.0001 respectively) and larval grazed (0.9%, *p* < 0.0001; 0.0%, *p* < 0.0001; and 0.1%, *p* < 0.0001; respectively) compared to fresh (40.5, 35.5 and 3.7% respectively) manure (Fig. S4 a, b, c). Interestingly, manure aging significantly influenced the abundance of Colpodellidae, where the relative abundance in aged was lower (2.8%,) compared to grazed (19.0%, *p* < 0.0001) and fresh (15.3%, *p* < 0.0001) manure (Fig. S4d). Relative abundances of the Euglyphida, Nolandellidae, Thecamoebidae and Oxytrichidae were greater in aged (7.7, 1.7, 0.2 and 29.9%, respectively) compared to both fresh (0.0%, *p* < 0.0001; 0.1%, *p* = 0.0003; 0.0%, *p* < 0.0001; and 0.2%, *p* = 0.001; respectively) and grazed (0.1%, *p* = 0.004; 0.0%, *p* = 0.015; 0.0%, *p* < 0.0001; and 0.4% *p* = 0.008, respectively) manure (Fig. S4 e, f, g, h). Also, the abundances of Chrysophyceae, Parabodonid and Vannellidae were greater in grazed (12.5, 28.8, 12.3% respectively) compared to aged (2.5%, *p* = 0.014; 6.3%, *p* = 0.014; 3.7%, *p* = 0.082, respectively) and fresh (0.2%, *p* < 0.0001; 0.3%, *p* < 0.0001; 0.4%, *p* < 0.0001; respectively) manure. Thraustochytriaceae were greater in grazed (14.9%) compared to fresh (0.2%, *p* < 0.0001) and lower compared to aged (16.8%, *p* = 0.73) manure.

At the lowest taxonomic levels, manure type significantly affected the abundance of dominant taxa (*p* < 0.05, Fig. 3c, S5). For instance, the relative abundance of *Buxtonella* (Ciliophora) and the genera of Metamonada: *Enteromonas*, *Trepomonas, Trimitus* and *Tetratrichomonas* (Fig. 3c) were significantly higher in fresh (35.5, 31.0, 5.0, 2.4 and 3.7%, respectively) compared to aged (0.0%, *p* < 0.0001; 0.3% *p* = 0.0001; 0.0%, *p* < 0.0001; 0.0%, *p* < 0.0001; and 0.0%, *p* < 0.0001, respectively) and grazed (0.0%, *p* < 0.0001; 0.4% *p* = 0.0004; 0.3%, *p* = 0.0003; 0.2%, *p* = 0.0002; 0.1%, *p* = 0.0002, respectively) manure. Although, the abundance of Ciliophora was higher in both fresh and aged manure, the genus *Buxtonella* was exclusively found in fresh while *Oxytricha* was found in aged manure (Fig. 3c). The relative abundance of *Oxytricha, Cercomonas*, unclassified Nolandellidae, unclassified Euglyphida and unclassified Thraustochytriaceae were greater in aged (29.8, 3.4, 1.7, 7.7 and 16.8%, respectively) compared to fresh (0.1%, *p* = 0.0005; 0.0%, *p* < 0.0001; 0.1%, *p* = 0.0003; 0.0%, *p* < 0.0001; and 0.2%, *p* < 0.0001, respectively) and grazed (0.4%, *p* = 0.014; 0.1%, *p* = 0.008; 0.1%, *p* = 0.015; 0.1%, *p* = 0.004; and 14.9%, *p* < 0.0001; respectively) manure. Interestingly, abundances of *Parabodo*, *Colpodella*, *Vannella*, unclassified Filosa-Sarcomonadea and unclassified Chrysophyceae were greater in grazed (28.8, 19.0, 12.3, 5.5 and 12.5% respectively) compared to fresh (0.3%, *p* < 0.0001; 15.3%, *p* = 0.15; 0.4%, *p* < 0.0001; 0.1%, *p* < 0.0001; 0.2%, *p* < 0.0001, respectively) and aged (6.3%, *p* = 0.014; 2.8%, *p* < 0.0001; 3.7%, *p* = 0.08; 3.2%, *p* = 0.26; 2.5%, *p* = 0.014, respectively) manure (Fig. 3c).

### Microbial diversity and community composition

**Fig. 4:**
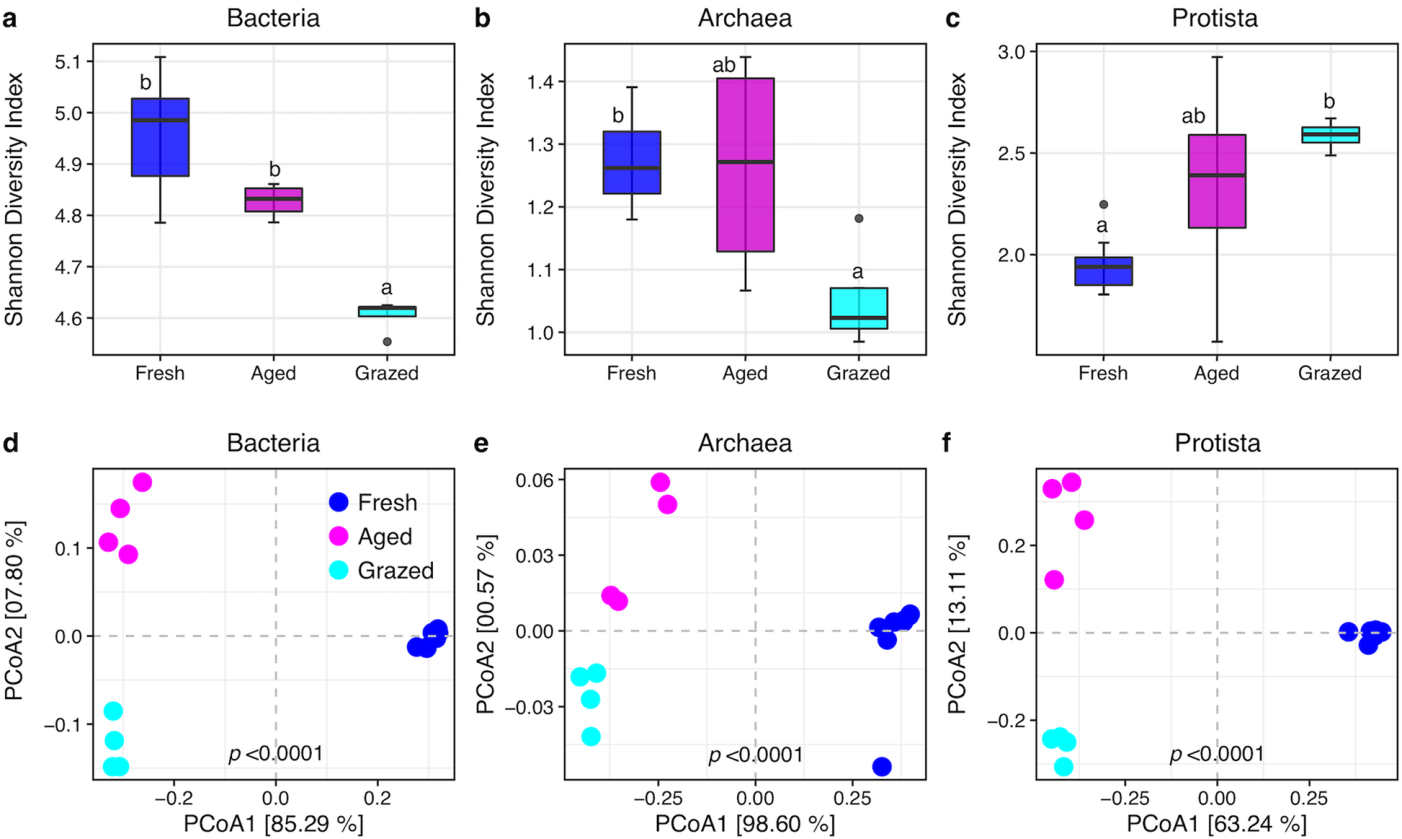
Microbial α-diversity (Shannon diversity index) and community compositions in dairy cattle manure before and after aging and house fly larval grazing. Top: Shannon diversity index in three manure types for **a**) Bacteria, **b**) Archaea, and **c**) Protist. In the box plots, median and interquartile range (25th–75th percentiles; boxes) with upper and lower range of values are shown. The different letters on top of each box indicate the significant differences between manure types (*p* ≤ 0.05). Each manure consisted of 4 replicates except fresh which included 8 replicates. Bottom: Principal Components Analysis (PCoA) illustrating Bray-Curtis distances in individual samples of Fresh, Aged, and Grazed manure types. Community composition for **d**) Bacteria, **e**) Archaea, and **f**) Protist are shown

Manure type significantly affected the archaeal Shannon (*p* = 0.018) and Simpson (*p* < 0.0001) diversity indices but not species richness (*p* = 0.15). Multiple comparisons of means showed that the archaeal Shannon diversity index of grazed (1.05) was significantly lower than fresh (1.27, *p* = 0.0180) but non-significantly lower than aged (1.26, *p* = 0.0530) (Fig. 4b, Table S3). The effect of manure type on bacterial Shannon diversity index (*p* < 0.0001), Simpson diversity index (*p* = 0.0007) and species richness (*p* < 0.0001) were significant. Multiple comparisons of means revealed that the bacterial Shannon diversity index was significantly lower in grazed (4.61) compared to fresh (4.96, *p* < 0.0001) and aged (4.83, *p* = 0.005) manure type (Fig. 4a, Table S3). Also, manure type significantly influenced the protist Shannon (*p* = 0.02) and Simpson (*p* = 0.03) diversity indices and species richness (*p* < 0.0001). Pairwise comparisons showed the protists Shannon diversity index was significantly greater in grazed (2.59) than fresh (0.76, *p* = 0.002) but non-significantly greater than aged (2.33, *p* = 0.14) manure (Fig. 4c, Table S3).

The overall patterns of bacterial (Fig. 4d, S6a, b), archaeal (Fig. 4e, S6c, d) and protist (Fig. 4f, S6e, f) community compositions among manure types were distinctly separated in the first two axes of the principal coordinates analysis based on Bray-Curtis, Uni-Frac, and Jaccard (binary) dissimilarity indices. Permutational multivariate analysis of variance revealed that those distinct patterns were statistically significant for bacterial (*p* < 0.0001), archaeal (*p* < 0.0001), and protist (*p* < 0.0001) communities (Table S4). Moreover, microbial community compositions also were well separated on canonical correspondence analysis, where manure properties (total carbon (TC), total nitrogen (TN) and carbon to nitrogen ratio (CN)) significantly correlated with the bacterial, archaeal and protist community compositions (Fig. S7, Table S5).

### Relationships of manure properties, microbial communities and diversity

The major bacterial phyla (Proteobacteria, Bacteroidetes) and protist phyla (Discoba, Lobosa, Ochrophyta and Stramenopiles) were negatively correlated with TN and TC but positively correlated with CN (Table S6). However, the bacterial phylum Firmicutes, and protist phyla Ciliophora and Metamonada were positively correlated with TN and TC (Table S6). In the lower taxonomic levels, significant positive correlations between TN and/or TC and bacterial taxa: *Acenetobacter*, *Alistipes, Bacteroides*, *Campylobacter*, *Clostridium* sensu stricto, Clostridiales unclassified, Lachnospiraceae unclassified, Ruminococcaceae unclassified, *Succinivibrio*, *Phascolarctobacterium*, *Acholeplasma* and *Tissierella*; archaeal genera: *Methanosphaera*, *Methanobacter*; and protist taxa: *Blastocystis*, *Buxtonella, Enteromonas*, Hexamitinae-Enteromonadida unclassified, *Trepomonas*, *Trimitus* and *Tetratrichomonas* (Table S7) were observed. Other bacterial taxa: *Pseudomonas*, unclassified Comamonadaceae unclassified, *Taibaiella*, Bacteroidetes unclassified, Sphingobacteriaceae unclassified, Acidaminococcaceae unclassified; archaeal genera: *Methanomassiliicoccus* and *Methanocorpusculum*; and protist taxa *Parabodo*, *Vannella*, Lobosa unclassified, Filosa-Sarcomonadea unclassified were negatively correlated with TN and TC (Table S7). The analysis of correlation showed that α-diversity indices for bacteria (Shannon and Species Richness) and archaea (Shannon, Simpson, Pielou’s Evenness) were positively correlated with TN and TC but bacteria (Simpson), and protist (Shannon and Species Richness) were negatively correlated with TN and TC (Table S8).

### House fly larval grazing changes the manure properties

In dry manure, TC content ranged from 87.3–127.9 g/kg, TN ranged from 9.2–17.2 g/kg and CN ranged from 6.72–10.22. There was strong correlation between TC, TN and CN, and statistically significant effects of manure types on TC (*F*_(2, 13)_ = 11.04, *p* = 0.0015), TN (*F*_(2, 13)_ = 113.6, *p* < 0.0001) and CN (*F*_(2, 13)_ = 64.96, *p* < 0.0001) were observed. Pairwise comparisons of means showed that TN was significantly lower in grazed compared to both fresh (*p* < 0.0001) and aged (*p* = 0.0058) manure (Fig. S8b) whereas TC was significantly lower in grazed compared to fresh manure (*p* = 0.0039, Fig. S8a), CN was significantly higher in grazed compared to both fresh (*p* < 0.0001) and aged (*p* < 0.0002) manure (Fig. S8c).

## Discussion

Animal manure serves as an optimal developmental substrate for house fly larvae due to its nutritional composition and live microbial community [1, 10, 12, 14]. Because house fly larvae ingest the microbes along with substrate, our aim was to assess changes in the manure microbial communities and their diversities after completion of house fly larval development, and to compare those changes to the starting microbial community and to similarly aged manure devoid of larvae. Previous studies have demonstrated that fresh manure contains high levels of organic matter, TN and active microbes [1, 4]. Manure decomposes as it ages, and biotic and abiotic factors change the quality of substrate, water content, pH and oxygen. The presence of house fly larvae during aging further modifies manure quality by utilizing available nutrients such as TN and phosphorus [1], by aerating the substrate microenvironment as they move in search of food, by excreting enzymes such as lysozymes and proteases [15] as well as antimicrobial peptides and by ingesting microbes. While larval grazing directly affects microbial communities and their diversity (discussed further below), our study revealed that changes to TC, TN and CN also significantly correlated to changes in bacterial, archaeal and protist communities and their compositions and diversities.

Overall, we found that the manure archaeal community composition and diversity was significantly affected by aging and larval grazing. Manure archaeal communities were represented by a single phylum Euryarchaeota, which is congruent with previous studies that reported Euryarchaeota as the dominant phylum of the archaeal communities in pig and cow manure [16, 17]. Although the archaeal community was represented by this single phylum, a finer taxonomic resolution revealed a variation in archaeal communities across the manure types. Both aging and larval grazing of manure altered the abundance of the genera *Methanomassiliicoccus* and *Methanosphaera*, suggesting that manure aging, irrespective of the presence of larvae, was the major factor influencing abundance of these taxa. The most abundant genera, *Methanobrevibacter* and *Methanocorpusculum,* were significantly influenced by manure age and house fly larval grazing. *Methanobrevibacter* was highly abundant in fresh manure likely because it is a major archaeon colonizing the rumen of herbivores [18–21]. House fly larval grazing decreased the relative abundance of genus *Methanocorpusculum* which was subsequently displaced by *Methanobrevibacter*. Manure aging similarly affected *Methanocorpusculum* abundance, but to a lower extent as seen with larval grazing. Multiple factors including aeration of the substrate by larvae, competing microbial activities, and/or direct larval feeding may have contributed to these changes.

Irrespective of manure type, bacterial communities were dominated by the phyla Proteobacteria, Firmicutes and Bacteroidetes which is in accordance with other studies that evaluated the bacterial communities during manure vermicomposting or composting [12, 13, 22, 23]. House fly larval grazing promoted the colonization of Bacteroidetes, specifically the families Flavobacteriaceae and Porphyromonadaceae. Larval grazing did not affect the abundance of Proteobacteria and Firmicutes, both predominant phyla in the rumen or gut of higher vertebrate animals [24–28]. However, incubation alone (aging, whether in the presence or absence of larvae) significantly altered their abundance. At finer taxonomic levels, fresh manure was dominated by several rumen-associated taxa such as Ruminococcaceae unclassified, *Ruminococcus*, *Succinivibrio*, Lachnospiraceae unclassified, *Phascolarctobacterium*, *Clostridium*, and *Alistipes* as has been previously reported [29]. *Ruminococcus* spp. decompose a wide range of polysaccharides and fiber [30], which supports their high abundance in fresh cow manure, which is rich in undigested polysaccharides. However, as aging and larval grazing changes manure quality, pH and oxygen content, an unsuitable environment would be created that would not support these anaerobes. House fly larval grazing also significantly affected abundance of *Bacteroides*, *Taibaiella*, *Flavobacterium*, *Pseudomonas*, *Sphingopyxis*, and *Sphigobacterium*. *Bacteroides* are anaerobes and commonly found in the gut of humans [31], animals [29, 32] or insects [33]. Because larval activity likely aerates the substrate, we expected anaerobe abundance to be greatly reduced in larval grazed manure. In contrast, the relative abundance of facultative anaerobes such as *Flavobacterium* (Bacteroidetes), increased significantly after house fly larval grazing. House fly larval grazing also significantly promoted other facultatively anaerobic Bacteroidetes such as *Taibaiella* and *Sphigobacterium* as well as *Sphingopyxis* (Proteobacteria) and *Pseudomonas* (Proteobacteria). Manure CN increased significantly in house fly grazed manure which could bolster fast-growing microbial communities, as the CN reflects the ability of microbes to utilize carbon and nitrogen for microbial processes such as decomposition of organic matter [34]. Moreover, the effect from larval symbionts, if any, in those communities would be negligible as our experiment started with house fly eggs. Furthermore, as the house fly larval gut is lined with peritrophic matrix, microbiota present therein are acquired from the substrate (e.g., manure) and are transient, having no access to the epithelial cells of the gut for colonization and establishment.

Interestingly, significantly low bacterial diversity (number of OTUs) in larval grazed manure compared to both aged and fresh suggest that house fly larvae feed on specific groups of live bacteria [9]. This result is in concordance with a previous study that demonstrated the bacterial diversity and richness significantly decrease during vermicomposting of swine manure by house fly larvae [13]. The reduction in bacterial diversity between fresh and aged manure could be due to a shift from communities of strict anaerobes to facultative anaerobes, which has been previously reported in a study of aging horse manure [35]. Such variability in diversity also was reflected in community composition as seen in clusters of samples within each manure type as in the PCoA and CCA plots.

Protist communities including the dominant phyla Ciliophora, Apicomplexa, Stamenopiles, Ochrophyta, Cercozoa, Metamonada, Discoba and Lobosa were significantly different across manure type. Manure aging appeared to be one of the major factors influencing the abundances of phyla Metamonada, Stramenopiles and Lobosa. Abundances of those phyla were changed drastically in both aged and fly larval grazed manure compared to fresh manure. Several members of the Metamonada are known obligate anaerobes who colonize the alimentary canal of animals [36]. Therefore, we infer that Metamonada in the fresh manure may have originated from cattle gut and survived several hours or, alternatively, that DNA from dead cells was detectable initially but degraded over time. Metamonada abundance was reflected by several genera, *Enteromonas*, *Trimitus*, *Tetratrichomonas* and *Treponomonas,* whose abundances all decreased with manure aging, irrespective of larval presence. Interestingly, in the grazed manure the overall abundance of Ciliophora was significantly lower. However, at lower taxonomic levels, Ciliophora was represented by two major genera: *Buxtonella* and *Oxytricha*. Furthermore, the abundance of *Buxtonella* severely decreased with manure age and the abundance of *Oxytricha* increased with aging, but only in the absence of larvae. Some species of *Buxtonella* are known pathogens of cattle commonly found in their gut and are obligate anaerobes [37], while several species of *Oxytricha* are free-living and frequently reported from different environments such as soil and water [38, 39]. The high abundance of *Oxytricha* in aged but not in grazed manure could be due to direct competition for bacteria by grazing larvae and/or the larvae feeding on the protists themselves. Alternatively, the high abundance of these two different genera of Ciliophora in either fresh or aged manure could be due to a bias introduced by primer selection, which can amplify only ~ 200 bp of the partial V7-V8 region of 18S rRNA gene. Even though the 18S rRNA gene is highly conserved, this partial sequence may not provide good resolution at the lower taxonomic levels [40]. House fly larval grazing also significantly influenced the relative abundance of phylum Cercozoa which increased with aging but decreased with larval grazing; this trend also was reflected at the lower taxonomic levels in *Cercomonas* and Euglyphida unclassified. A previous study reported that in the rhizosphere, addition of phosphorus and nitrogen to the soil increases the abundance of α-Proteobacteria which is a high-quality food for *Cercomonas* [41]. It follows that larval grazing on manure results in depletion of various nutrients and subsequently groups of bacteria that are optimal food for *Cercomonas,* which would indirectly result in reduced abundance; however, as with the ciliates, house fly larvae also may directly ingest the cercozoans. The relative abundance of other phyla Apicomplexa, Ochrophyata, Discoba and Lobosa were greater in house fly larval grazed manure compared to fresh or aged. Interestingly, observation at a finer taxonomic resolution revealed that one or more genera (or taxa) drove the overall patterns at the phylum level. For example, the relative abundance of *Colpodella* (Apicomplexa), Chrysophyceae unclassified (Ochrophyta), *Parabodo* (Discoba) and *Vannella* (Lobosa) were similar to their phyla abundances. Many species of *Colpodella* are known to feed on other protists [42] which may have made them better competitors and predators of other taxa in the manure. Also, the feeding behavior of different groups of protists differs. For instance, members of the genus *Vannella* are raptorial feeders which use pseudopods to feed on both free living and surface attached bacteria [43]. This feeding behavior could have benefited *Vannella* to predate on a wider range of bacteria that are available in the substrate while in the presence of house fly larvae. Therefore, the high abundance of those taxa in house fly larval grazed manure is likely attributable to their feeding behavior.

Protist community composition and diversity were affected by both larval grazing and aging of the manure. Unlike bacterial diversity, larval grazing promoted the protist diversity. The inverse relationship of protist diversity and bacterial diversity is likely due to different behavior and feeding habits of those organisms. For example, many bacteria are prey of protists [44], insect larvae [8, 9] and other organisms. Our method of conducting the experiment in a controlled growth chamber, which provides a favorable environment for colonization of dormant and low abundant species, could influence the increased diversity of protists in aged and grazed manure. Consequently, an increase in biomass of individual species contributed to the template for PCR which directly influenced abundances of microbial communities and diversity.

Total carbon and nitrogen levels in dairy cattle manure decreased over time, suggesting the active role of microorganisms in decomposition of complex organic matter present in the manure and utilization of decomposed products as carbon and energy sources [3–5]. Besides microbial activity, presence of house fly larvae in the manure further reduced manure TC and TN by 17 and 38% respectively. Reduction of nutrient levels in manure after house fly larval grazing is in accordance with previous studies that demonstrated TN, TC and other nutrients levels decreased in cattle manure used to rear larvae [1, 2]. Changes after grazing also suggest that microbes are active in manure containing larvae, since this ratio reflects the ability of microbes to utilize carbon and nitrogen during decomposition [34].

Competition and interactions between members of an ecosystem, including predator-prey, antagonistic and symbiotic relationships among organisms, play a major role on microbial community compositions and diversity. For example, protozoa feed on variety of bacteria [45], protozoa and archaea or develop a symbiotic relationship with them [46]. Antagonistic relationships among organisms also are seen in microcosm environments where some organisms, for example *Pseudomonas fluorescens,* produce secondary metabolites that protect bacteria from protozoan predators [47] and other organisms. Both house fly larvae and protists feed on bacteria in the substrate, the competition for food and space could lead to the altered microbial communities, their compositions and diversities after house fly larval grazing in the manure. While only the contribution of house fly larvae was measured in our study, the complex interactions and contributions of other predators, competitors and promoters of members of the microbial community deserves further investigation in future studies.

The results presented in this study have some limitations. The larval resource was artificially finite by being constrained to a pan and having no further influx of material or microbes, which does not reflect manure usage for development in the natural setting. Because the experiment was conducted once, the variability in fecal microbial inputs in different time may not be reflected as cattle fecal/rumen microbial communities can be influenced by various abiotic and biotic factors such as diet, climate variables, cattle age, and others. Also, our study utilized the bacterial 16S rRNA primer pairs to characterize archaeal communities, which resulted in a low number of sequences assigned to archaea that could limit the broader assessment of those taxa. This study also utilized primer pairs for V4 and V7-V8 region of 16S and 18S rRNA genes, respectively which can amplify < 300 bp fragments. Even though both 16S and 18S rRNA genes are highly conserved, partial sequence of ribosomal genes may not resolve at the lower taxonomic resolution [40, 48]. Pairing sequencing-based studies with microbial culture or metagenomics, as well as including intermittent time points from egg inoculation to pupation, could give more insight into the complex changes that house fly larval grazing imparts on the manure microbial community.

## Conclusions

We characterized the microbial communities of dairy cattle manure and described the changes to those communities both over time as manure aged and in the presence of grazing house fly larvae. Even though aging significantly affected the diversity and abundance of bacterial, archaeal and protist communities, house fly grazing over time on manure significantly altered the diversity and abundances of various taxa, most notably a decrease in abundances of Bacteroidetes, Ciliophora, Metamonada, Cercozoa and genera within those phyla. Over time, both microbial activity and house fly larval grazing utilized available nutrients such as TN and TC in the substrate, subsequently altering the quality of organic matter that in turn significantly affected the composition and diversity of bacteria, archaea and protist communities. These results provide insight into the role of house fly larval grazing on manure microbial communities, including their abundance and diversity. Further studies are required to understand the role of these altered communities in health, fitness and development of house fly larvae which have potential applications towards developing novel larval control methods.

## Methods

### Assay design

A microcosm experiment was designed to examine the effects of house fly larval grazing on dairy cattle manure microbial community and diversity. The assay consisted of three manure types: fresh, aged, and grazed. Fresh dairy cattle feces (hereafter manure, ~ 4 kg) was collected from the barn floor at the Dairy Teaching and Research Center at Kansas State University, Manhattan, KS, USA. Manure was collected with a sterile spatula at a depth of at least 0.5 cm, avoiding the outer layer, in order to reduce environmental contamination, and was placed in a 4-L Ziploc bag for transport. The manure was stored in a sterile 10-L plastic container covered with sterile pillowcase at room temperature overnight at the laboratory. After ~ 16 h, manure was homogenized using a sterile spatula and 150 g was distributed into each of sixteen sterile 0.25-L plastic containers. Eight containers were immediately processed (see below) and served as “fresh” samples (four matched for each of the manure types). For “aged” manure type, four containers were placed into individual sterile 10-L secondary plastic bins and enclosed within sterile pillowcases. For “grazed” manure type, each container of manure received seventy-five house fly eggs (i.e. 1 egg/2 g manure). Eggs were obtained from the breeding colony reared on dairy cattle manure [49] at Kansas State University, where house fly rearing condition was tempearture 28 +/− 2 °C, day/night 16/8 h and humidity ~ 50%. Similar to aged manure containers, each of the larval grazed containers were placed into secondary plastic bins and enclosed within sterile pillowcases. All containers were incubated at 28 +/− 1 °C and day/night 16/8 h in a growth chamber (Percival Scientific Inc., USA). The grazed containers that had been inoculated with house fly eggs were monitored daily until all larvae had pupated (10 days), after which pupae were aseptically collected. On average 69% of the total eggs developed to pupae and 63% of the total eggs successfully metamorphosed to adults (Table S1). Manure from both aged and grazed groups (*n* = 4, each) was collected on day 10 for processing as described below.

### Collection of manure samples and physicochemical analysis

Fresh manure pans (*n* = 8) were homogenized immediately after setup and 2 ml from each pan was removed and immediately stored at − 80 °C until DNA extraction (see below). For physicochemical analysis, 50 g of manure homogenate was collected from each of the 8 containers and samples were stored at − 20 °C until further analysis. For the aged and larval grazed containers (n = 4, each), after 10 d incubation, manure was homogenized, and samples were collected for DNA extraction and physicochemical analyses as above. All 50 g manure samples were sent to the Ward Laboratories, Inc., Kearney, NE (https://www.wardlab.com) on ice packs for total organic carbon and nitrogen analyses.

### DNA extraction, amplicon library preparation, sequencing and data analysis

Total genomic DNA was extracted from 200 mg of individual manure samples using the QIAamp PowerFecal DNA kit (QIAGEN, USA) according to the manufacturer’s instructions. Isolates from laboratory cultures of the bacterium *Citrobacter freundii* and fungus *Cladosporium* sp. isolates were processed similarly as manure samples and served as control for bacterial and eukaryal DNA, respectively. The concentration and quality of sample DNA were determined by NanoDrop (Thermo Fisher Scientific, USA) and agarose gel electrophoresis, respectively. DNA was stored at − 20 °C until sequence library preparation.

Primer pairs for bacterial 16S rRNA gene (515F, [50] and 806R, [51]) and eukaryotic 18S rRNA gene (V7-V8, [52]) with Illumina MiSeq adapters were used for PCR. PCR assays with each pair of primer sets were performed in duplicate for DNA samples. Positive controls for each primer pair consisted of bacterial DNA (*Citrobacter freundii*, 16S rRNA primers) and fungal DNA (*Cladosporium* sp., 18S rRNA primers) from laboratory cultures. Duplicate reactions with PCR grade water as template served as negative controls. Each PCR reaction (25 μl) contained a final concentration of 1× KAPA HiFi HotStart ReadyMixPCR Kit (Kapa Biosystems, Wilmington, MA, USA), 0.2 μM each primer and 12.5 ng DNA template (or water for negative control). The PCR was performed in a DNA Engine® Thermal Cycler (Bio-Rad, Hercules, CA, USA), with the following reaction conditions: 95 °C for 3 min followed by 25 cycles of 95 °C for 30 s, 55 °C for 30 s and 72 °C for 30 s, and 72 °C for 5 min. Agarose gel electrophoresis (1% in TAE) was performed to confirm amplification. Equal volumes of duplicate PCR products were pooled. Further, library preparation and sequencing were performed following 16S metagenomic sequencing library preparation protocol for Illumina MiSeq system (https://tinyurl.com/ybxgxsqm) at the Department of Plant and Environmental Sciences, Clemson University, Clemson, SC, USA. The raw sequence data were deposited at the National Center for Biotechnology Information Sequence Read Archive under the BioProject number PRJNA701469.

Raw sequence reads were processed in the mothur bioinformatic software pipeline (version 1.39.5, [53]). For 16S rRNA amplicons, paired-end sequence reads were assembled, and primers were removed. Low quality (q < 25) sequence reads with ambiguous base, ambiguous length (> 280 bp) and > 6 homopolymers were removed. Further, high quality sequence reads were aligned to the SILVA reference alignment database [54] using the Needleman-Wunsch global alignment method [55] and unaligned sequences were removed. Chimeric sequences were checked using VSEARCH [56] and were removed. Non-chimeric sequence reads with sequence similarity of 97% were clustered into operational taxonomic units (OTUs). Naïve Bayesian Classifier [57] and RDP reference database [58] were used to determine the consensus taxonomy of each OTU. OTUs classified as unknown, eukaryota, cyanobacteria, mitochondria and chloroplast were removed. Further, low abundant (< 2) and erroneous OTUs (OTUs in bacterial control sample that were classified other than *Citrobacter* sp.) were determined and removed from all samples. The resulting OTU table was further normalized to account for differences in sequence depths among samples by subsampling to equal sequence depth per sample. The normalized bacterial OTU table contained 334,610 sequence reads per sample and normalized archaeal OTU table contained 1597 sequence reads per sample.

For 18S rRNA amplicons, primers, low quality and erroneous bases (> 200) were removed using cutadapt [59]. High-quality, paired-end sequences were analyzed as described above for 16S rRNA amplicon except that quality filter ambiguous length was < 200 bp. Consensus taxonomy was determined using Naïve Bayesian Classifier [57] with the protist ribosomal reference database (PR^2^ [60];). OTUs classified as eukaryote_unclassified, fungi, archaeplastida, and metazoa which represented 32% of total sequences were removed. Low abundant and erroneous OTUs (OTUs in fungal control sample that were classified other than *Cladosporium* sp.) were determined and removed. Further, to minimize the bias due to sequencing depth, the final OTU table was prepared using 13,437 sequence reads per sample. The final OTUs data were used for downstream statistical analysis.

### Statistical analyses

Statistical analyses were performed in R statistical programming environment (version 3.4.4, [61]). To determine the effect of manure types on manure physicochemical properties TC, TN and CN, analysis of variance (ANOVA) followed by a pairwise post hoc Tukey Honest Significant Differences (TukeyHSD) test was performed to compare the group means between manure types. A rarefaction curve was generated and estimated the OTU richness of each sample in the vegan package in R (version 2.5–3, [62]). OTUs were summarized at different taxonomic levels: phyla, family and genus and the effect of manure types on the abundance of most abundant phyla, family or genus were determined using ANOVA or Kruskal Wallis test (if the variable did not meet the assumption of equal variance). We chose most abundant genera if relative abundance of a taxon was > 1% in at least one sample or present in one manure types. A pairwise post hoc comparison of means was performed as described above to determine the differences between manure types. Further, to evaluate the variation in the abundance of taxa between samples, Z-scores were calculated for individual taxa at their finest taxonomic level (hereafter taxon) across the samples. Using OTU data, diversity indices: Shannon (*H′*), Simpson, species richness and Pielou’s evenness were calculated in the vegan package in R (version 2.5–3, [62]). To investigate the effects of larval grazing or aging on diversity indices ANOVA followed by pairwise post hoc Tukey HSD for multiple comparisons of group means were performed. Principal coordinate analyses (PCoA) based on Bray-Curtis (abundance), Uni-Frac (phylogenetic) or Jaccard (binary) distances were performed to investigate the microbial (bacterial, archaeal and protist) community composition in each pair of samples. To examine the differences in community composition among manures, permutation analysis of variance (PERMANOVA) was performed. To assess the effects of manure properties (TC, TN and CN) on microbial community compositions, canonical correspondence analysis (CCA) followed by permutation-based ANOVA was performed. Pearson correlation coefficients were calculated to evaluate the relationship of microbial α-diversity indices, most abundant taxa and major phyla with manure properties (TC, TN and CN). All statistical tests with *p*-value < 0.05 were considered statistically significant.

## Supplementary Information

**Additional file 1.**

**Additional file 2.**

## Data Availability

The datasets that support the conclusions of this study are available in NCBI SRA (National Center for Biotechnology Information Sequence Read Archive) under the accession number PRJNA701469, within the article and in the additional files.

## Abbreviations

rRNA: Ribosomal RNA
TC: Total Carbon
TN: Total Nitrogen
CN: Total Carbon to Total Nitrogen Ratio
OTU: Operational Taxonomic Unit
CCA: Canonical Correspondence Analysis
PCoA: Principal Coordinates Analysis
ANOVA: Analysis of Variance
PERMANOVA: Permutation Analysis of Variance
