# Supplementary material for: House fly larval grazing alters dairy cattle manure microbial communities
Source: BMC Microbiol. 2021 Dec 15;21:346. doi: 10.1186/s12866-021-02418-5 (PMC8672618; doi:10.1186/s12866-021-02418-5)

**Supplementary Figures**

**House Fly Larval Grazing Alters Dairy Cattle Manure Microbial Communities**

Saraswoti Neupane^1*^, Christopher Saski^2^, Dana Nayduch^3^

^1^Department of Entomology, Kansas State University, Manhattan, KS, USA

^2^Department of Plant and Environmental Sciences, Clemson University, Clemson, SC, USA

^3^USDA-ARS, Center for Grain and Animal Health Research, Arthropod-Borne Animal Diseases Research Unit, Manhattan, KS, USA

**Fig. S1:** Effects of manure type on the relative abundance of archaeal families. The plots show the relative abundance of families: a) Methanobacteriaceae, b) Methanocorpusculaceae, c) Methanomassiliicoccaceae in Fresh, Aged, and Grazed manure. In the box plots, median and interquartile range (25^th^-75^th^ percentiles; boxes) with upper and lower range of values are shown.

Effect of manure types was determined using Kruskal-Wallis test followed by pairwise Wilcoxon tests. Statistically significant differences in relative abundance between manure types (*, *p* <0.05; **, *p* < 0.01) and non-significant (ns). Each manure type consisted of 4 replicates except Fresh which included 8 replicates.

**Fig. S2:** Effect of manure types on the relative abundance of the most abundant bacterial families. The plots show the relative abundance of: a) Clostridiaceae, b) Lachnospiraceae, c) Ruminococcaceae, d) Erysipelotrichaceae, e) Veillonellaceae, f) Bacteroidaceae, g) Prevotellaceae, h) Flavobacteriaceae, i) Porphyromonadaceae, j) Moraxellaceae, k) Succinivibrionaceae, l) Aeromonadaceae, m) Sphingobacteriaceae, n) Comamonadaceae, o) Pseudomonadaceae, and p) Xanthomonadaceae in Fresh, Aged, and Grazed manure. In the box plots, median and interquartile range (25^th^-75^th^ percentiles; boxes) with upper and lower range of values are shown. Effect of manure types was determined using Kruskal-Wallis test followed by pairwise Wilcoxon tests. Statistically significant differences in relative abundance between manure types (*, *p* <0.05; **, *p* < 0.01) and non-significant (ns). Each manure type consisted of 4 replicates except Fresh which included 8 replicates.

**Fig. S3:** Profiling of most abundant bacterial taxa classified at the finest taxonomic resolution in the individual manure replicate samples. The heat map shows the z-score values of the abundance of each bacterial taxon in each sample displayed by color intensity as in color bar. Sample ID: Fresh-1 to Fresh-8 are Fresh, Aged-1 to Aged-4 are Aged and Grazed-1 to Grazed-4 are house fly larval grazed manure. Taxa names with the same color belong to the same phylum.

**Fig. S4:** Effect of manure type on the relative abundance of protist taxa. The plots show the relative abundance of: a) Hexamitinae-Enteromonadida, b) Trichostomatia, c) Trichomonadidae, d) Colpodellidae, e) Euglyphida, f) Nolandellidae, g) Thecamoebidae, h) Oxytrichidae, i) Chrysophyceae, j) Parabodonid, k) Thraustochytriaceae and l) Vannellidae, in Fresh, Aged, and Grazed manure. In the box plots, median and interquartile range (25^th^-75^th^ percentiles; boxes) with upper and lower range of values are shown. Effect of manure types was determined using Kruskal-Wallis test followed by pairwise Wilcoxon tests. Statistically significant differences in relative abundance between manure types (*, *p* <0.05; **, *p* < 0.01) and non-significant (ns). Each manure type consisted of 4 replicates except Fresh which included 8 replicates.

**Fig. S5:** Profiling of the most abundant protist taxa classified at the finest taxonomic resolution in individual manure replicate samples. The heat map shows the row z-score values of the abundance of each protist taxon in each sample displayed by color intensity as in color bar. Sample ID: Fresh-1 to Fresh-8 are Fresh, Aged-1 to Aged-4 are Aged and Grazed-1 to Grazed-4 are house fly larval grazed manure. Taxa names with same color belong to the same phylum.

**Fig. S6:** Microbial community composition in dairy cattle manure. Principal Components Analysis (PCoA) illustrating a), c), e) Uni-Frac; b), d), f) Jaccard (binary) distances between individual samples of Fresh, Aged, and house fly larval grazed (Grazed) manure. Communities are: a) and b) bacterial, c) and d) archaeal, and e) and f) protist.

**Fig. S7:** Relationships of microbial community composition and manure properties. Canonical Correspondence Analysis (CCA) demonstrating relationships between bacterial, archaeal and protist communities to total carbon (TC), total nitrogen (N), and carbon to nitrogen ratio (CN) in Fresh, Aged and house fly larval Grazed manure.

**Fig. S8**: Dairy cattle manure properties before and after aging and house fly larval grazing. a) total carbon, b) total nitrogen, and c) carbon to nitrogen ratio in three manure types: Fresh, Aged, and Grazed. Each manure type consisted of 4 replicates except fresh which included 8 replicates. In the box plots, median and interquartile range (25^th^-75^th^ percentiles; boxes) with upper and lower range of values are shown. The different letters on top of each box indicate the significant differences between manure types (P ≤ 0.05).

**Fig. S1**


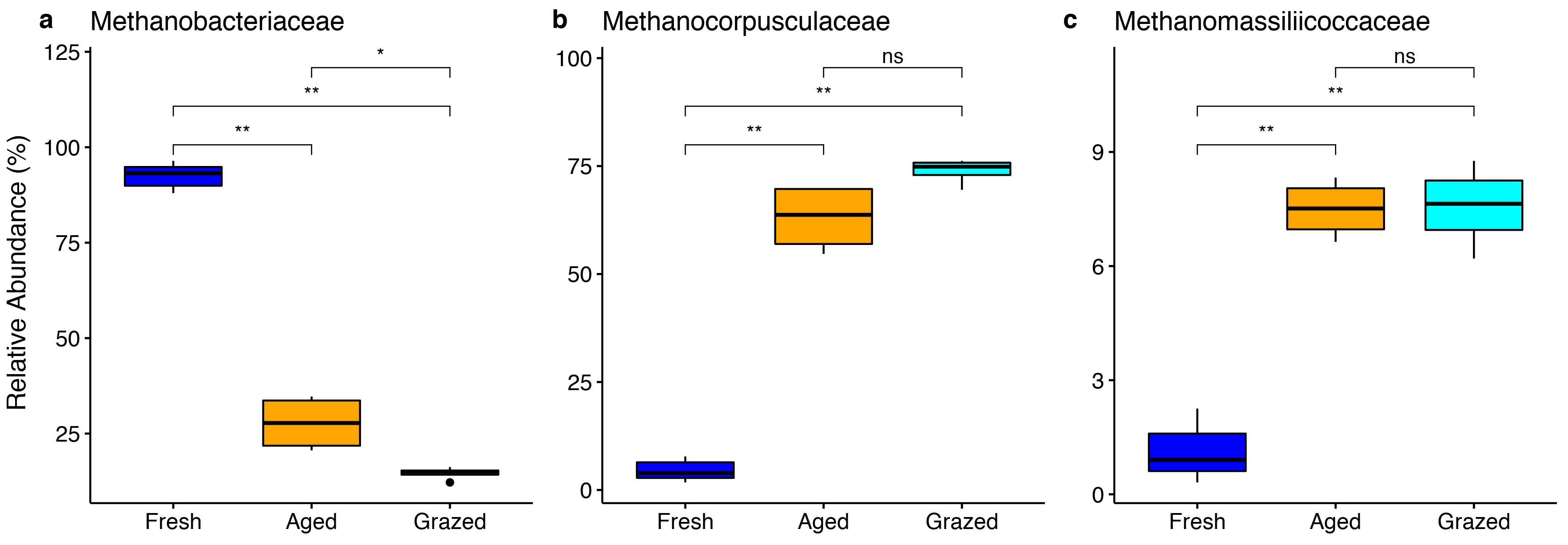


**Fig. S2**
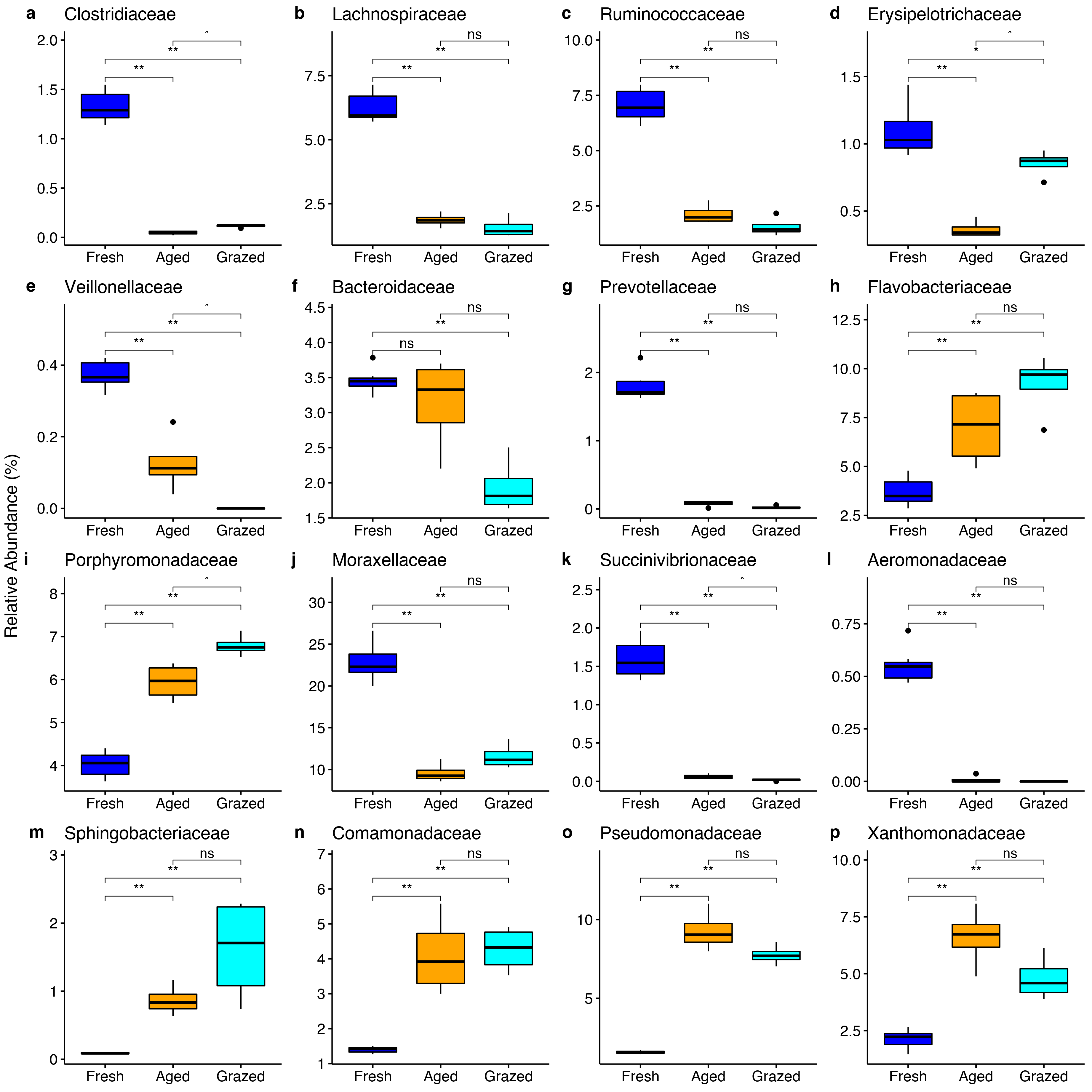


**Fig. S3**
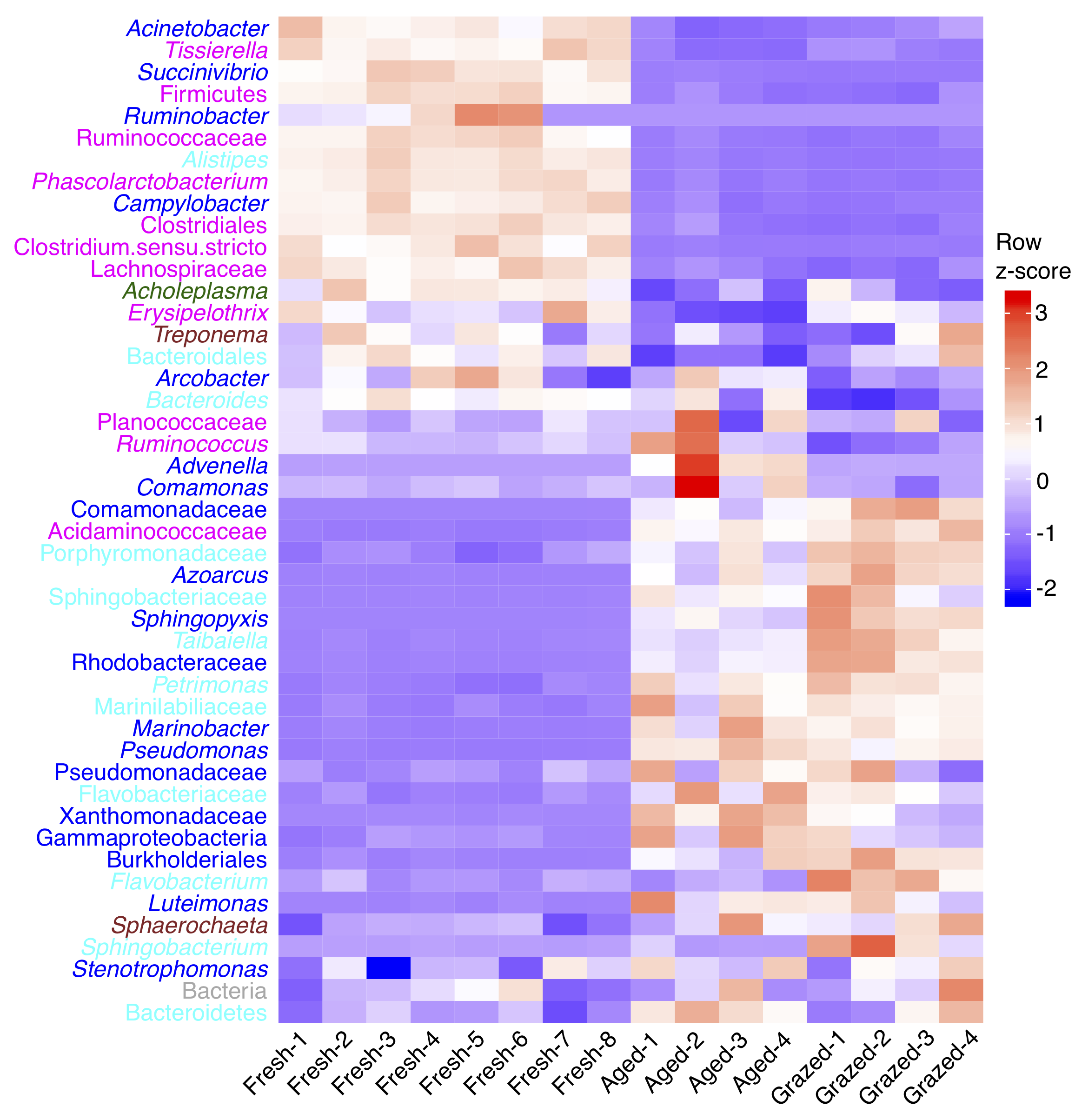


**Fig. S4**
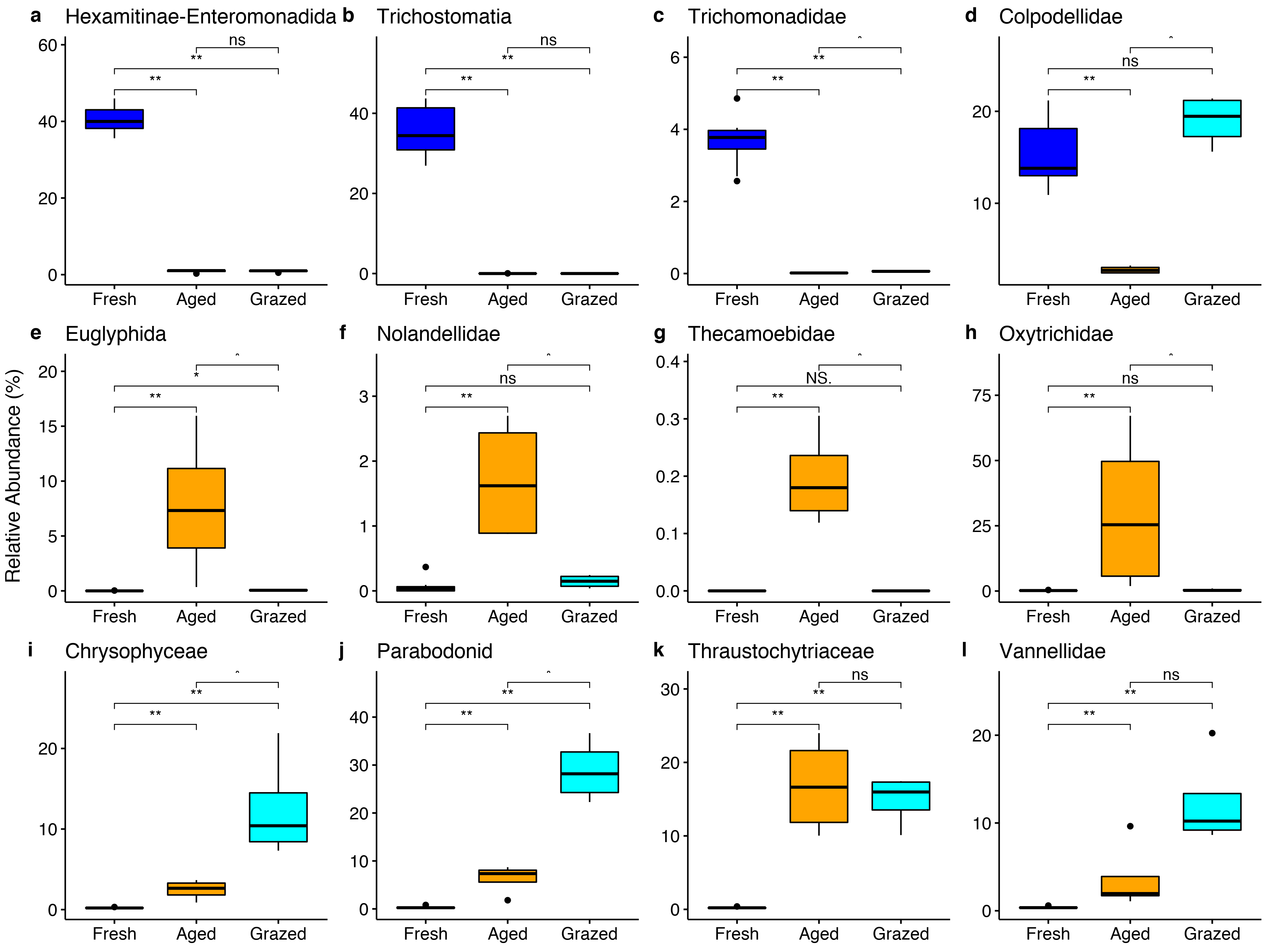


**Fig. S5**
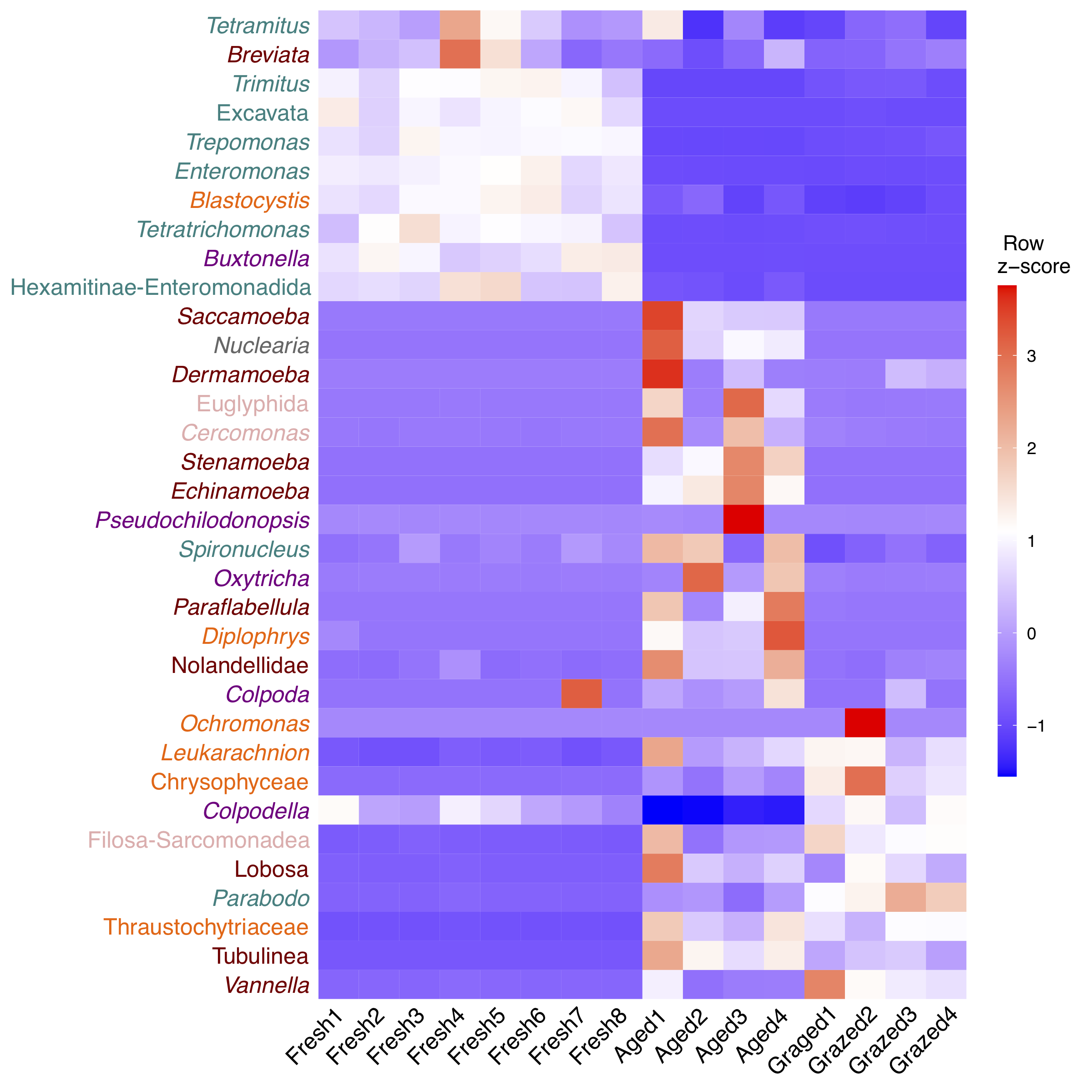


**Fig. S6**


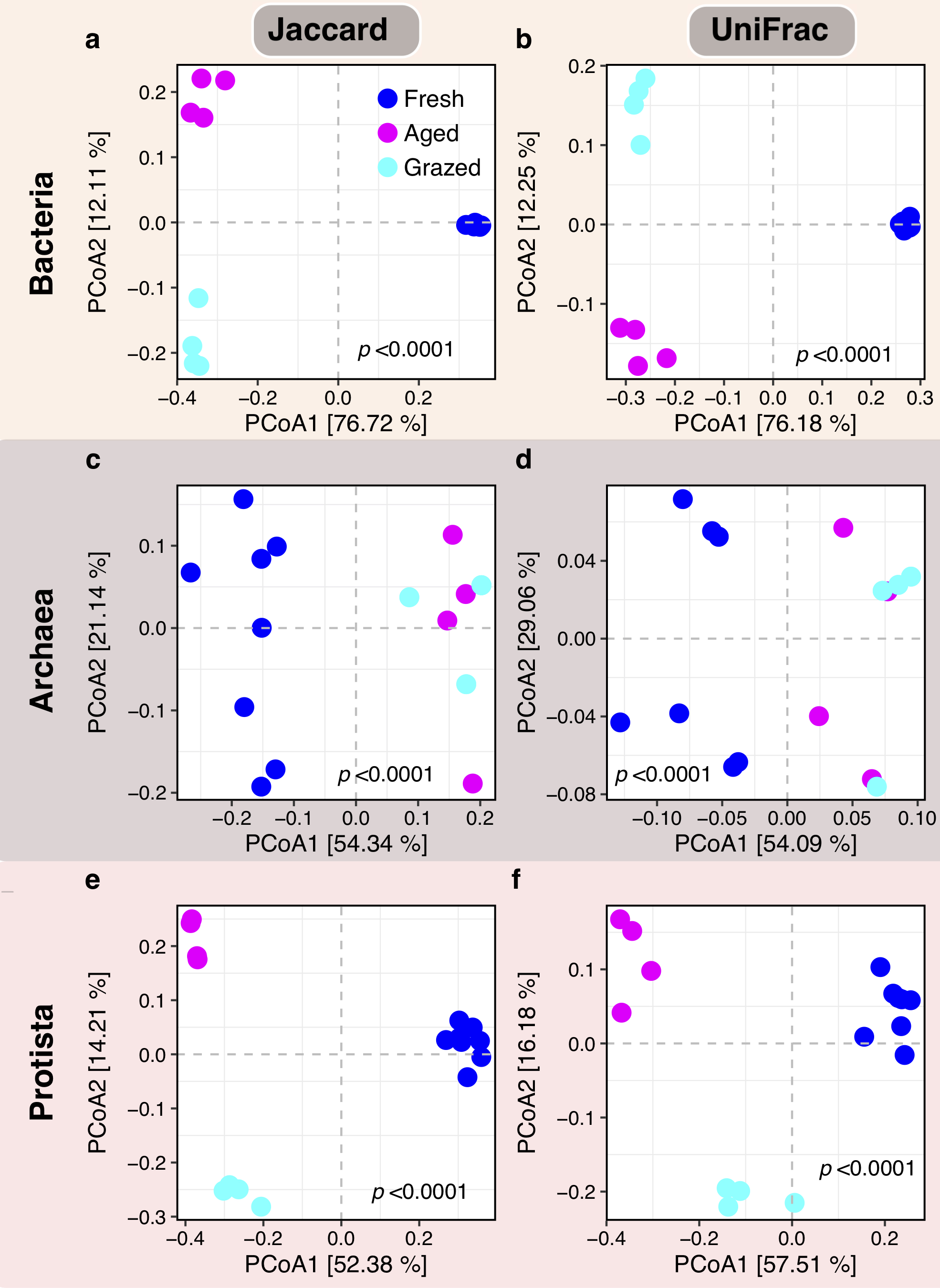


**Fig. S7**


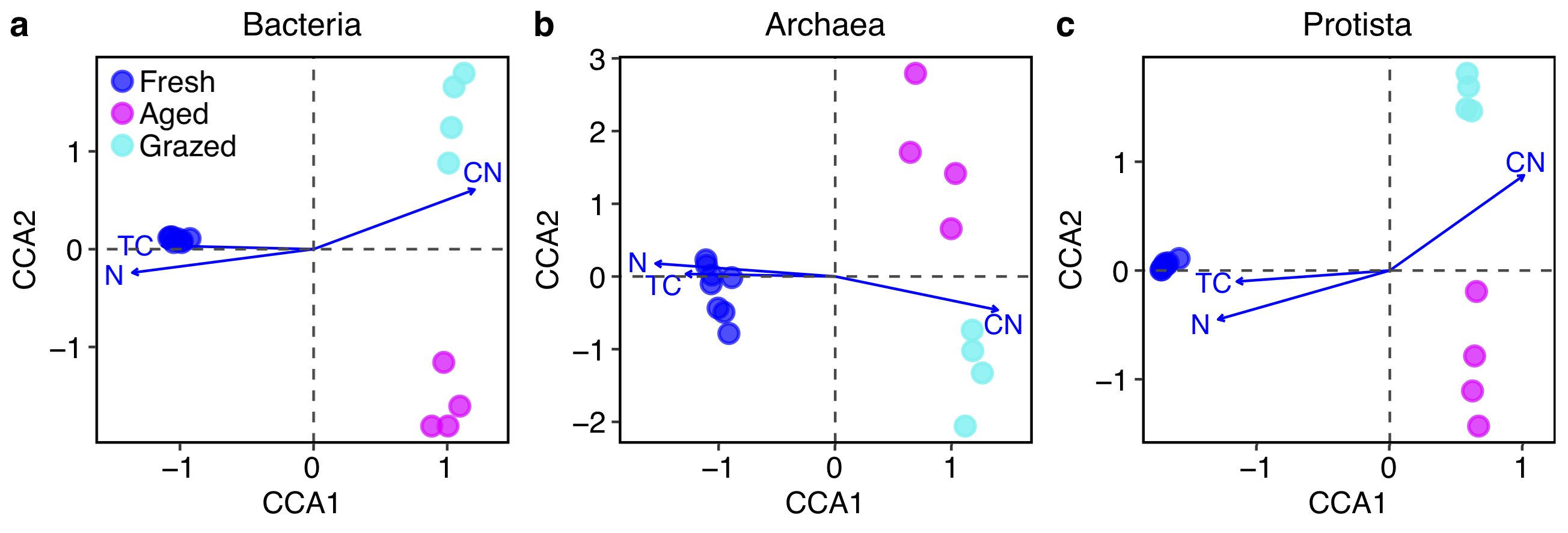


**Fig. S8**


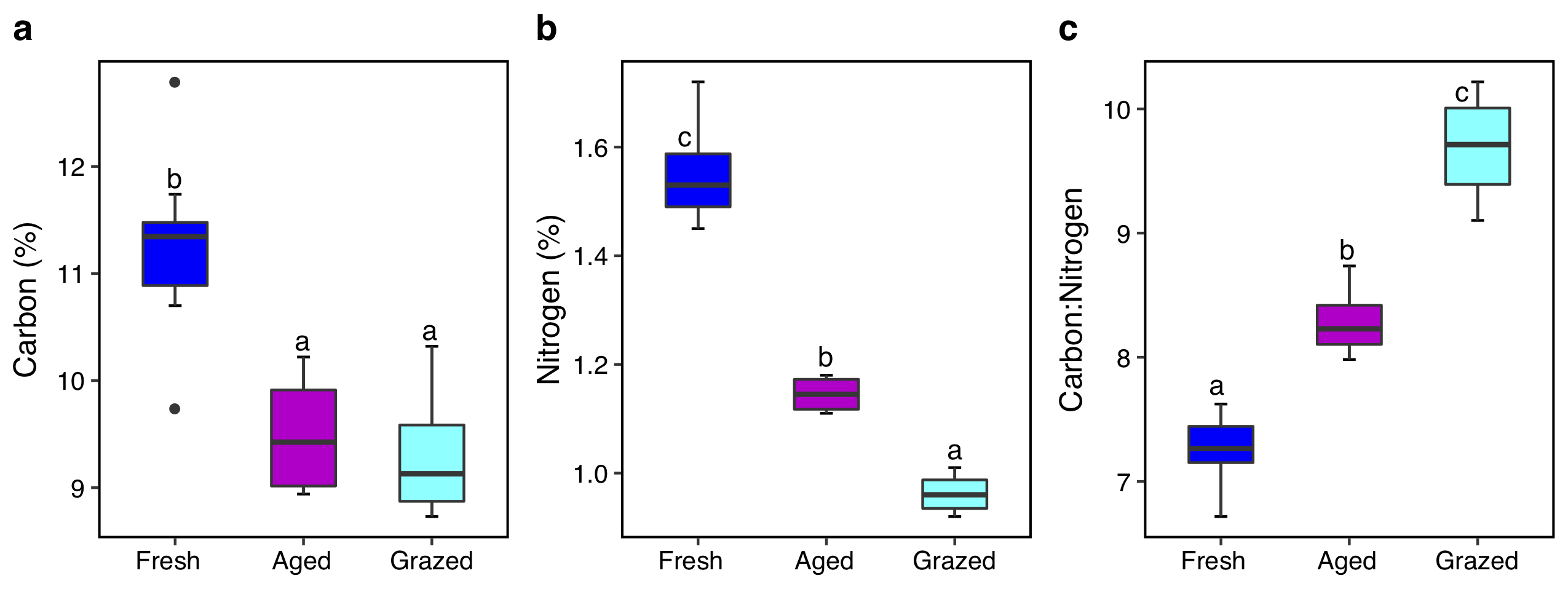

Supplement: Supplementary file 1 — Additional file 1. [file 12866_2021_2418_MOESM1_ESM.docx]
