# Supplementary material for: House fly larval grazing alters dairy cattle manure microbial communities
Source: BMC Microbiol. 2021 Dec 15;21:346. doi: 10.1186/s12866-021-02418-5 (PMC8672618; doi:10.1186/s12866-021-02418-5)
Supplement: Supplementary file 2 — Additional file 2. [file 12866_2021_2418_MOESM2_ESM.docx]

**Supplementary Tables**

**House Fly Larval Grazing Alters Dairy Cattle Manure Microbial Communities**

Saraswoti Neupane^1*^, Christopher Saski^2^, Dana Nayduch^3^

^1^Department of Entomology, Kansas State University, Manhattan, KS, USA

^2^Department of Plant and Environmental Sciences, Clemson University, Clemson, SC, USA

^3^USDA-ARS, Center for Grain and Animal Health Research, Arthropod-Borne Animal Diseases Research Unit, Manhattan, KS, USA

**Table S1:** House fly larval survival in manure.

**Table S2:** Effects of house fly larval grazing on manure bacterial, archaeal and protist taxa at their lowest taxonomic level.

**Table S3:** Manure bacterial, archaeal and protist alpha diversities.

**Table S4**: Effect of manure types on manure bacterial, archaeal and protist community composition determined by PERMANOVA/Adonis.

**Table S5**: Relationships of manure bacterial, archaeal and protist community composition and manure quality.

**Table S6:** Correlation between selected microbial phyla and manure properties. Pearson correlation coefficients (r) between bacterial and protist major phyla and total nitrogen (TN), total carbon (TC) and carbon nitrogen ratio (CN).

**Table S7:** Correlation between selected microbial taxa and manure properties. Pearson correlation coefficients (r) between bacterial, archaeal, eukaryal taxa in the lowest taxonomic resolution and total nitrogen (TN), total carbon (TC) and carbon nitrogen ratio (CN).

**Table S8:** Correlation between α-Diversities and manure properties. Pearson correlation coefficients (r) between bacterial, archaeal, protist α-diversity and total nitrogen (TN), total carbon (TC) and carbon nitrogen ratio (CN).

**Table S1:** House fly larval survival in manure.

| **Replicate** | **No of eggs** | **No. of Pupae** | **No of Emerged** |
| --- | --- | --- | --- |
| **R1** | 75 | 57 | 51 |
| **R2** | 75 | 60 | 56 |
| **R3** | 75 | 56 | 52 |
| **R4** | 75 | 34 | 30 |

**Table S2:** Effects of house fly larval grazing on manure bacterial, archaeal and protist taxa at their lowest taxonomic level.

|  | **Taxa** | **Effect of Manure types** | **Fresh vs Aged** | **Fresh vs Grazed** | **Aged vs Grazed** |
| --- | --- | --- | --- | --- | --- |
| **Bacteria** | *Acinetobacter* | <0.0001 | <0.0001 | <0.0001 | 0.2830 |
|  | *Pseudomonas* | <0.0001 | <0.0001 | <0.0001 | 0.0292 |
|  | Ruminococcaceae unclassified | <0.0001 | <0.0001 | <0.0001 | 0.9003 |
|  | *Ruminococcus* | 0.0033 | 0.0476 | 0.0952 | 0.0024 |
|  | *Arcobacter* | 0.2290 | 0.9219 | 0.3020 | 0.2517 |
|  | *Campylobacter* | <0.0001 | <0.0001 | <0.0001 | 0.8948 |
|  | *Ruminobacter* | 0.0238 | 0.0542 | 0.0542 | 1.0000 |
|  | *Succinivibrio* | <0.0001 | <0.0001 | <0.0001 | 0.9247 |
|  | *Marinobacter* | <0.0001 | <0.0001 | <0.0001 | 0.7246 |
|  | Comamonadaceae unclassified | <0.0001 | 0.0002 | <0.0001 | 0.0012 |
|  | *Comamonas* | 0.0270 | 0.0513 | 0.7717 | 0.0326 |
|  | *Advenella* | 0.0001 | 0.0001 | 0.9456 | 0.0007 |
|  | Rhodobacteraceae unclassified | <0.0001 | <0.0001 | <0.0001 | 0.0002 |
|  | *Azoarcus* | <0.0001 | <0.0001 | <0.0001 | 0.0026 |
|  | Lachnospiraceae unclassified | <0.0001 | <0.0001 | <0.0001 | 0.5284 |
|  | Clostridiales unclassified | <0.0001 | <0.0001 | <0.0001 | 0.3523 |
|  | *Phascolarctobacterium* | <0.0001 | <0.0001 | <0.0001 | 0.7091 |
|  | Planococcaceae unclassified | 0.4970 | 0.5072 | 0.9999 | 0.5901 |
|  | *Sphaerochaeta* | 0.0090 | 0.0475 | 0.0140 | 0.8369 |
|  | *Treponema* | 0.3050 | 0.2762 | 0.8079 | 0.6790 |
|  | *Acholeplasma* | 0.0008 | 0.0010 | 0.0138 | 0.4555 |
|  | *Luteimonas* | <0.0001 | <0.0001 | 0.0013 | 0.5618 |
|  | Pseudomonadaceae unclassified | 0.0345 | 0.0402 | 0.1633 | 0.7681 |
|  | *Stenotrophomonas* | 0.1630 | 0.1874 | 0.3661 | 0.9166 |
|  | Xanthomonadaceae unclassified | <0.0001 | <0.0001 | 0.0014 | 0.0006 |
|  | Gammaproteobacteria unclassified | 0.0002 | 0.0001 | 0.0296 | 0.0505 |
|  | *Taibaiella* | <0.0001 | <0.0001 | <0.0001 | <0.0001 |
|  | *Erysipelothrix* | <0.0001 | <0.0001 | 0.6811 | 0.0011 |
|  | Flavobacteriaceae unclassified | 0.00011 | 0.0002 | 0.0025 | 0.4013 |
|  | *Flavobacterium* | <0.0001 | 1.00 | <0.0001 | <0.0001 |
|  | *Petrimonas* | <0.0001 | <0.0001 | <0.0001 | 0.2819 |
|  | Porphyromonadaceae unclassified | <0.0001 | 0.0003 | <0.0001 | 0.0017 |
|  | Bacteria unclassified | 0.4370 | 0.8475 | 0.4075 | 0.7852 |
|  | Bacteroidetes unclassified | 0.0068 | 0.0053 | 0.2371 | 0.1926 |
|  | *Bacteroides* | <0.0001 | 0.4542 | 0.0001 | 0.0028 |
|  | Bacteroidales unclassified | <0.0001 | <0.0001 | 0.7585 | 0.0045 |
|  | Marinilabiliaceae unclassified | <0.0001 | <0.0001 | <0.0001 | 0.9351 |
|  | *Tissierella* | <0.0001 | <0.0001 | <0.0001 | 0.2825 |
|  | *Clostridium.sensu.stricto* | <0.0001 | <0.0001 | <0.0001 | 0.9991 |
|  | *Alistipes* | <0.0001 | <0.0001 | <0.0001 | 0.6444 |
|  | *Sphingopyxis* | <0.0001 | 0.0001 | <0.0001 | 0.0002 |
|  | Burkholderiales unclassified | <0.0001 | 0.0003 | <0.0001 | 0.0244 |
|  | Firmicutes unclassified | <0.0001 | <0.0001 | <0.0001 | 0.8203 |
|  | Acidaminococcaceae unclassified | <0.0001 | <0.0001 | <0.0001 | 0.0080 |
|  | Sphingobacteriaceae unclassified | <0.0001 | 0.0006 | <0.0001 | 0.4660 |
|  | *Sphingobacterium* | 0.0002 | 0.9289 | 0.0002 | 0.0011 |
| **Archaea** | *Methanimicrococcus* | 0.1680 | 0.7569 | 0.3033 | 0.1615 |
|  | *Methanosarcina* | 0.6220 | 0.9418 | 0.5946 | 0.8368 |
|  | *Methanomassiliicoccus* | <0.0001 | <0.0001 | <0.0001 | 0.9940 |
|  | *Methanosphaera* | <0.0001 | <0.0001 | <0.0001 | 0.4609 |
|  | *Methanocorpusculum* | <0.0001 | <0.0001 | <0.0001 | 0.0099 |
|  | *Methanobrevibacter* | <0.0001 | <0.0001 | <0.0001 | 0.0013 |
| **Protist** | *Blastocystis* | 0.0024 | <0.0001 | <0.0001 | 0.0823 |
|  | *Breviata* | 0.0663 | 0.1005 | 0.0966 | 0.7348 |
|  | *Buxtonella* | 0.0029 | <0.0001 | <0.0001 | 0.7709 |
|  | *Cercomonas* | 0.0035 | <0.0001 | 0.012 | 0.0204 |
|  | Chrysophyceae unclassified | 0.0017 | <0.0001 | <0.0001 | 0.0142 |
|  | *Colpoda* | 0.0574 | 0.0479 | 0.7109 | 0.0884 |
|  | *Colpodella* | 0.0063 | 0.0034 | 0.0558 | <0.0001 |
|  | *Dermamoeba* | 0.0038 | <0.0001 | 0.0023 | 0.1556 |
|  | *Diplophrys* | 0.0018 | <0.0001 | 0.4778 | <0.0001 |
|  | *Echinamoeba* | 0.0007 | <0.0001 | 1 | <0.0001 |
|  | *Enteromonas* | 0.0032 | <0.0001 | 0.0005 | 0.4065 |
|  | Euglyphida unclassified | 0.0023 | <0.0001 | 0.0049 | 0.0049 |
|  | Excavata unclassified | 0.002 | <0.0001 | <0.0001 | 0.5 |
|  | Filosa-Sarcomonadea unclassified | 0.003 | <0.0001 | <0.0001 | 0.2631 |
|  | Hexamitinae-Enteromonadida unclassified | 0.002 | <0.0001 | <0.0001 | 0.0617 |
|  | *Leukarachnion* | 0.0031 | <0.0001 | <0.0001 | 0.4007 |
|  | Lobosa unclassified | 0.0025 | <0.0001 | <0.0001 | 0.5365 |
|  | Nolandellidae unclassified | 0.0083 | 0.001 | 0.1338 | 0.0239 |
|  | *Nuclearia* | 0.0007 | <0.0001 | 1 | <0.0001 |
|  | *Ochromonas* | 0.2231 | 1 | 0.2472 | 0.2472 |
|  | *Oxytricha* | 0.0096 | 0.0015 | 0.1969 | 0.0223 |
|  | *Parabodo* | 0.0017 | <0.0001 | <0.0001 | 0.0142 |
|  | *Paraflabellula* | 0.0016 | <0.0001 | 0.1425 | <0.0001 |
|  | *Pseudochilodonopsis* | 0.3623 | 0.4956 | 0.8157 | 0.4956 |
|  | *Saccamoeba* | 0.0007 | <0.0001 | 1 | <0.0001 |
|  | *Spironucleus* | 0.0175 | 0.1992 | 0.0154 | 0.007 |
|  | *Stenamoeba* | 0.0007 | <0.0001 | 1 | <0.0001 |
|  | *Tetramitus* | 0.0278 | 0.0505 | 0.0231 | 0.5149 |
|  | *Tetratrichomonas* | 0.0017 | <0.0001 | <0.0001 | 0.0142 |
|  | Thraustochytriaceae unclassified | 0.0035 | <0.0001 | <0.0001 | 0.7846 |
|  | *Trepomonas* | 0.0017 | <0.0001 | <0.0001 | 0.0139 |
|  | *Trimitus* | 0.0016 | <0.0001 | <0.0001 | 0.0107 |
|  | *Tubulinea* | 0.0007 | <0.0001 | <0.0001 | <0.0001 |
|  | *Vannella* | 0.0024 | <0.0001 | <0.0001 | 0.0823 |

**Table S3:** Manure bacterial, archaeal and protist alpha diversities*.

|  | Alpha Diversity | Fresh Manure | Aged Manure | Grazed Manure |
| --- | --- | --- | --- | --- |
| Bacteria | Species Richness | 810^b^ (79) | 611^a^ (29) | 507^a^ (42) |
|  | Shannon diversity Index | 4.96^b^ (0.11) | 4.83^b^ (0.03) | 4.61^a^ (0.03) |
|  | Simpson diversity Index | 0.96^a^ (0.01) | 0.99^b^ (0.00) | 0.97^b^ (0.00) |
|  | Pielou's Evenness | 0.74^a^ (0.02) | 0.75^a^ (0.01) | 0.74^a^ (0.01) |
| Archaea | Species Richness | 12^a^ (1) | 13^a^ (1) | 13^a^ (1) |
|  | Shannon diversity Index | 1.27^b^ (0.08) | 1.26^ab^ (0.18) | 1.05^a^ (0.09) |
|  | Simpson diversity Index | 0.64^c^ (0.02) | 0.56^b^ (0.08) | 0.44^a^ (0.04) |
|  | Pielou's Evenness | 0.52^b^ (0.03) | 0.49^ab^ (0.06) | 0.42^a^ (0.03) |
| Protist | Species Richness | 58^a^ (12) | 116^c^ (13) | 84^b^ (16) |
|  | Shannon diversity Index | 1.96^a^ (0.14) | 2.33^ab^ (0.58) | 2.59^b^ (0.08) |
|  | Simpson diversity Index | 0.76^a^ (0.03) | 0.76^a^ (0.16) | 0.86^a^ (0.02) |
|  | Pielou's Evenness | 0.49^a^ (0.03) | 0.49^a^ (0.12) | 0.59^a^ (0.03) |

*The number presented in the table are mean of each manure types and the standard deviations are listed in parenthesis. Letters (a, b, c) following mean indicate statistically significant difference determined by linear model or Kruskal-Wallis test followed by pairwise comparisons using *post hoc* TukeyHSD test or Wilcoxon test.

**Table S4**: Effect of manure types on manure bacterial, archaeal and protist community composition determined by PERMANOVA/Adonis.

|  |  | **Pseudo F** | **R^2^** | ***P*-value** |
| --- | --- | --- | --- | --- |
|  | **Main Effects** |  |  |  |
| **Bacteria** | Manure types | 81.46 | 0.93 | <0.0001 |
| **Archaea** | Manure types | 367.43 | 0.98 | <0.0001 |
| **Protist** | Manure types | 20.16 | 0.76 | <0.0001 |

**Table S5**: Relationships of manure bacterial, archaeal and protist community composition and manure quality.

|  | **Main Effect** | **Chi Square** | **F** | **Pr(>F)** |
| --- | --- | --- | --- | --- |
| **Bacteria** | TN | 0.11 | 7.96 | 0.004 |
|  | TC | 0.09 | 6.40 | 0.009 |
|  | CN | 0.10 | 7.31 | 0.005 |
| **Archaea** | TN | 0.03 | 6.98 | 0.012 |
|  | TC | 0.02 | 4.38 | 0.05 |
|  | CN | 0.02 | 3.93 | 0.063 |
| **Protist** | TN | 0.41 | 5.89 | 0.001 |
|  | TC | 0.34 | 4.88 | 0.001 |
|  | CN | 0.42 | 6.03 | 0.001 |

TC = total carbon; TN = total nitrogen; CN = ratio of total carbon to total nitrogen.

**Table S6:** Correlation between selected microbial phyla and manure properties. Pearson correlation coefficients (r) between bacterial and protist major phyla and total nitrogen (TN), total carbon (TC) and carbon nitrogen ratio (CN).

|  | **Phylum** | **TN** | **TC** | **CN** |
| --- | --- | --- | --- | --- |
| **Bacteria** | Bacteroidetes | -0.96 | -0.76 | 0.92 |
|  | Firmicutes | 0.94 | 0.79 | -0.84 |
|  | Proteobacteria | -0.80 | -0.63 | 0.72 |
|  | Spirochaetes | -0.39 | -0.58 | 0.12 |
|  | Tenericutes | 0.66 | 0.55 | -0.55 |
| **Protist** | Apicomplexa | 0.14 | 0.30 | 0.15 |
|  | Cercozoa | -0.55 | -0.62 | 0.29 |
|  | Ciliophora | 0.55 | 0.42 | -0.61 |
|  | Discoba | -0.83 | -0.62 | 0.87 |
|  | Lobosa | -0.79 | -0.69 | 0.69 |
|  | Metamonada | 0.93 | 0.75 | -0.84 |
|  | Ochrophyta | -0.74 | -0.44 | 0.89 |
|  | Stramenopiles | -0.84 | -0.75 | 0.69 |

**Table S7:** Correlation between selected microbial taxa and manure properties. Pearson correlation coefficients (r) between bacterial, archaeal, eukaryal taxa in the lowest taxonomic resolution and total nitrogen (TN), total carbon (TC) and carbon nitrogen ratio (CN).

|  | **Taxon** | **TN** | **TC** | **CN** |
| --- | --- | --- | --- | --- |
| **Bacteria** | *Acholeplasma* | 0.66 | 0.56 | -0.54 |
|  | Acidaminococcaceae unclassified | -0.95 | -0.78 | 0.87 |
|  | *Acinetobacter* | 0.91 | 0.83 | -0.73 |
|  | *Advenella* | -0.32 | -0.34 | 0.13 |
|  | *Alistipes* | 0.94 | 0.77 | -0.85 |
|  | *Arcobacter* | 0.25 | 0.03 | -0.43 |
|  | *Azoarcus* | -0.93 | -0.73 | 0.91 |
|  | Bacteria unclassified | -0.38 | -0.56 | 0.12 |
|  | Bacteroidales unclassified | 0.36 | 0.29 | -0.28 |
|  | *Bacteroides* | 0.74 | 0.53 | -0.81 |
|  | Bacteroidetes unclassified | -0.54 | -0.70 | 0.21 |
|  | Burkholderiales unclassified | -0.89 | -0.62 | 0.95 |
|  | *Campylobacter* | 0.94 | 0.79 | -0.82 |
|  | Clostridiales unclassified | 0.94 | 0.74 | -0.87 |
|  | *Clostridium.sensu.stricto* | 0.93 | 0.80 | -0.81 |
|  | Comamonadaceae *unclassified* | -0.91 | -0.68 | 0.93 |
|  | *Comamonas* | -0.04 | -0.01 | -0.02 |
|  | *Erysipelothrix* | 0.42 | 0.57 | -0.11 |
|  | Firmicutes unclassified | 0.93 | 0.72 | -0.87 |
|  | Flavobacteriaceae unclassified | -0.73 | -0.53 | 0.71 |
|  | *Flavobacterium* | -0.71 | -0.45 | 0.85 |
|  | Gammaproteobacteria unclassified | -0.67 | -0.67 | 0.46 |
|  | Lachnospiraceae unclassified | 0.93 | 0.74 | -0.87 |
|  | *Luteimonas* | -0.79 | -0.66 | 0.69 |
|  | Marinilabiliaceae unclassified | -0.87 | -0.79 | 0.72 |
|  | *Marinobacter* | -0.87 | -0.77 | 0.73 |
|  | *Petrimonas* | -0.95 | -0.78 | 0.86 |
|  | Phascolarctobacterium | 0.93 | 0.77 | -0.84 |
|  | Planococcaceae unclassified | -0.07 | 0.04 | 0.14 |
|  | Porphyromonadaceae unclassified | -0.93 | -0.72 | 0.91 |
|  | Pseudomonadaceae unclassified | -0.55 | -0.36 | 0.57 |
|  | *Pseudomonas* | -0.89 | -0.80 | 0.72 |
|  | Rhodobacteraceae unclassified | -0.93 | -0.68 | 0.96 |
|  | *Ruminobacter* | 0.59 | 0.33 | -0.65 |
|  | Ruminococcaceae unclassified | 0.92 | 0.72 | -0.86 |
|  | *Ruminococcus* | 0.16 | 0.00 | -0.37 |
|  | *Sphaerochaeta* | -0.74 | -0.80 | 0.50 |
|  | Sphingobacteriaceae unclassified | -0.87 | -0.65 | 0.87 |
|  | *Sphingobacterium* | -0.67 | -0.37 | 0.86 |
|  | *Sphingopyxis* | -0.92 | -0.70 | 0.94 |
|  | *Stenotrophomonas* | -0.47 | -0.35 | 0.41 |
|  | *Succinivibrio* | 0.92 | 0.75 | -0.83 |
|  | *Taibaiella* | -0.92 | -0.67 | 0.97 |
|  | *Tissierella* | 0.91 | 0.84 | -0.72 |
|  | *Treponema* | 0.25 | 0.01 | -0.39 |
|  | Xanthomonadaceae unclassified | -0.67 | -0.61 | 0.51 |
| **Archaea** | *Methanimicrococcus* | -0.23 | -0.07 | 0.40 |
|  | *Methanobrevibacter* | 0.96 | 0.79 | -0.88 |
|  | *Methanocorpusculum* | -0.96 | -0.79 | 0.87 |
|  | *Methanomassiliicoccus* | -0.94 | -0.82 | 0.80 |
|  | *Methanosarcina* | -0.21 | -0.24 | 0.14 |
|  | *Methanosphaera* | 0.94 | 0.82 | -0.82 |
| **Protist** | *Blastocystis* | 0.93 | 0.74 | -0.87 |
|  | *Breviata* | 0.47 | 0.36 | -0.46 |
|  | *Buxtonella* | 0.91 | 0.79 | -0.79 |
|  | *Cercomonas* | -0.31 | -0.45 | 0.03 |
|  | Chrysophyceae unclassified | -0.71 | -0.41 | 0.88 |
|  | *Colpoda* | -0.01 | 0.03 | 0.00 |
|  | *Colpodella* | 0.15 | 0.31 | 0.13 |
|  | *Dermamoeba* | -0.34 | -0.46 | 0.10 |
|  | *Diplophrys* | -0.25 | -0.21 | 0.14 |
|  | *Echinamoeba* | -0.33 | -0.43 | 0.06 |
|  | *Enteromonas* | 0.93 | 0.75 | -0.84 |
|  | Euglyphida unclassified | -0.30 | -0.44 | 0.02 |
|  | Excavata unclassified | 0.95 | 0.81 | -0.82 |
|  | Filosa-Sarcomonadea unclassified | -0.84 | -0.72 | 0.78 |
|  | Hexamitinae-Enteromonadida unclassified | 0.89 | 0.78 | -0.77 |
|  | *Leukarachnion* | -0.82 | -0.68 | 0.74 |
|  | Lobosa unclassified | -0.67 | -0.60 | 0.54 |
|  | Nolandellidae unclassified | -0.34 | -0.38 | 0.13 |
|  | *Nuclearia* | -0.31 | -0.42 | 0.06 |
|  | *Ochromonas* | -0.29 | 0.00 | 0.52 |
|  | *Oxytricha* | -0.20 | -0.15 | 0.13 |
|  | *Parabodo* | -0.83 | -0.62 | 0.88 |
|  | *Paraflabellula* | -0.29 | -0.30 | 0.12 |
|  | *Pseudochilodonopsis* | -0.18 | -0.31 | -0.03 |
|  | *Saccamoeba* | -0.29 | -0.39 | 0.05 |
|  | *Spironucleus* | -0.10 | -0.14 | -0.04 |
|  | *Stenamoeba* | -0.32 | -0.40 | 0.08 |
|  | *Tetramitus* | 0.55 | 0.34 | -0.61 |
|  | *Tetratrichomonas* | 0.89 | 0.70 | -0.82 |
|  | Thraustochytriaceae unclassified | -0.86 | -0.76 | 0.71 |
|  | *Trepomonas* | 0.92 | 0.77 | -0.81 |
|  | *Trimitus* | 0.91 | 0.74 | -0.80 |
|  | Tubulinea unclassified | -0.71 | -0.66 | 0.53 |
|  | *Vannella* | -0.78 | -0.58 | 0.84 |

**Table S8:** Correlation between α-Diversities and manure properties. Pearson correlation coefficients (r) between bacterial, archaeal, protist α-diversity and total nitrogen (TN), total carbon (TC) and carbon nitrogen ratio (CN).

| **Domain** | **α- Diversity** | **TN** | **TC** | **CN** |
| --- | --- | --- | --- | --- |
| **Bacteria** | Shannon Diversity Index | 0.76 | 0.43 | -0.91 |
|  | Simpson Diversity Index | -0.77 | -0.83 | 0.50 |
|  | Species Richness | 0.88 | 0.67 | -0.87 |
|  | Pielou's Evenness | -0.22 | -0.48 | -0.10 |
| **Archaea** | Shannon Diversity Index | 0.50 | 0.27 | -0.62 |
|  | Simpson Diversity Index | 0.81 | 0.58 | -0.85 |
|  | Species Richness | -0.40 | -0.48 | 0.22 |
|  | Pielou's Evenness | 0.64 | 0.45 | -0.70 |
| **Protist** | Shannon Diversity Index | -0.72 | -0.65 | 0.62 |
|  | Simpson Diversity Index | -0.42 | -0.35 | 0.42 |
|  | Species Richness | -0.62 | -0.51 | 0.51 |
|  | Pielou's Evenness | -0.53 | -0.52 | 0.44 |
